# A panomics-driven framework for the improvement of major food legume crops: advances, challenges, and future prospects

**DOI:** 10.1093/hr/uhaf091

**Published:** 2025-03-18

**Authors:** Hongliang Hu, Xingxing Yuan, Dinesh Kumar Saini, Tao Yang, Xinyi Wu, Ranran Wu, Zehao Liu, Farkhandah Jan, Reyazul Rouf Mir, Liu Liu, Jiashun Miao, Na Liu, Pei Xu

**Affiliations:** Zhejiang-Israel Joint Laboratory for Plant Metrology and Equipment Innovation, College of Life Sciences, China Jiliang University, Hangzhou 310018, China; Institute of Industrial Crops, Jiangsu Academy of Agricultural Sciences, Nanjing 210014, China; Department of Plant and Soil Science, Texas Tech University, Lubbock, TX 79409, USA; State Key Laboratory of Crop Gene Resources and Breeding/ Institute of Crop Sciences, Chinese Academy of Agricultural Sciences, Haidian District, Beijing 100081, China; State Key Laboratory for Managing Biotic and Chemical Threats to the Quality and Safety of Agro-products, Institute of Vegetables, Zhejiang Academy of Agricultural Sciences, Hangzhou, China; Institute of Industrial Crops, Jiangsu Academy of Agricultural Sciences, Nanjing 210014, China; State Key Laboratory of Crop Gene Resources and Breeding/ Institute of Crop Sciences, Chinese Academy of Agricultural Sciences, Haidian District, Beijing 100081, China; Division of Genetics & Plant Breeding, Faculty of Agriculture, SKUAST-Kashmir, Wadura Campus, Sopore, Jammu and Kashmir 193201, India; Centre for Crop and Food Innovation, WA State Agricultural Biotechnology Centre, Murdoch University, Murdoch WA 6150, Australia; Zhejiang Xianghu Laboratory, Hangzhou, China; Zhejiang Xianghu Laboratory, Hangzhou, China; Zhejiang Xianghu Laboratory, Hangzhou, China; State Key Laboratory for Quality and Safety of Agro-products, Institute of Vegetables, Zhejiang Academy of Agricultural Sciences, Hangzhou, China; Zhejiang-Israel Joint Laboratory for Plant Metrology and Equipment Innovation, College of Life Sciences, China Jiliang University, Hangzhou 310018, China

## Abstract

Food legume crops, including common bean, faba bean, mungbean, cowpea, chickpea, and pea, have long served as vital sources of energy, protein, and minerals worldwide, both as grains and vegetables. Advancements in high-throughput phenotyping, next-generation sequencing, transcriptomics, proteomics, and metabolomics have significantly expanded genomic resources for food legumes, ushering research into the panomics era. Despite their nutritional and agronomic importance, food legumes still face constraints in yield potential and genetic improvement due to limited genomic resources, complex inheritance patterns, and insufficient exploration of key traits, such as quality and stress resistance. This highlights the need for continued efforts to comprehensively dissect the phenome, genome, and regulome of these crops. This review summarizes recent advances in technological innovations and multi-omics applications in food legumes research and improvement. Given the critical role of germplasm resources and the challenges in applying phenomics to food legumes—such as complex trait architecture and limited standardized methodologies—we first address these foundational areas. We then discuss recent gene discoveries associated with yield stability, seed composition, and stress tolerance and their potential as breeding targets. Considering the growing role of genetic engineering, we provide an update on gene-editing applications in legumes, particularly CRISPR-based approaches for trait enhancement. We advocate for integrating chemical and biochemical signatures of cells (‘molecular phenomics’) with genetic mapping to accelerate gene discovery. We anticipate that combining panomics approaches with advanced breeding technologies will accelerate genetic gains in food legumes, enhancing their productivity, resilience, and contribution to sustainable global food security.

## Introduction

Legume crops, members of the Fabaceae (or Leguminosae) family, are essential to global agriculture. The family consists of approximately 800 genera and 20 000 species [1], and is traditionally divided into three major subfamilies: Papilionoideae, Caesalpinioideae, and Mimosoideae. The majority of economically significant legumes, such as peas, soybeans, and lentils, belong to the Papilionoideae subfamily. Key staples in global agriculture include soybeans (*Glycine max* L.), common beans (*Phaseolus vulgaris* L.), and peas (*Pisum sativum* L.). Other important legumes, such as faba beans (*Vicia faba* L.), cowpeas (*Vigna unguiculata* L. Walp), chickpeas (*Cicer arietinum* L.), and mungbeans (*Vigna radiata* L.), are dietary staples in regional cuisines.

**Figure 1:**
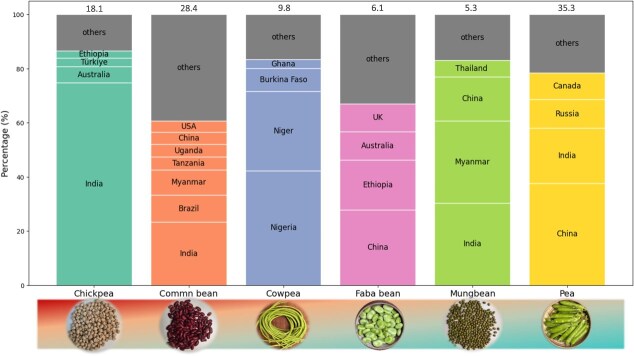
Global yield percentage of major food legumes by country. Value on top of each column represents the crop’s global production (million tons), respectively. The global production data for chickpea (dry), common bean (dry), cowpea (dry), faba bean (dry), and pea (dry and fresh) are sourced from FAOSTAT 2022 (https://www.fao.org/faostat/en/#data/QCL). Mungbean is not listed in the statistical database of FAO, so its data is sourced from Nair *et al*. [2].

Today, food legumes are grown worldwide, particularly in Africa, Australia, North America, India, and other Asian countries [2] (Fig. 1). They are ranked as the second most cultivated crop group, following cereals [3]. Legumes are popular for their numerous advantages, including high and affordable protein content, as well as their ecological importance in nitrogen fixation [4, 5]. However, climate change, driving challenges such as waterlogging, flooding, and biotic stressors, poses a significant threat to food security in key legume-producing regions [6]. Despite the extensive global cultivation of legumes, their yield per hectare remains considerably lower than that of cereals. This yield gap highlights the urgent need to identify novel genes that influence yield and environmental resilience to develop more effective improvement strategies.

In recent decades, significant progress has been made in food legume improvement. Advances in phenotyping and sequencing technologies, alongside the development of various bioinformatic tools, have greatly enhanced omics-based plant research. This research, which includes phenomics, genomics, transcriptomics, metabolomics, and proteomics [7], has been instrumental in identifying gene networks and metabolic pathways associated with desirable traits [8]. These advances have facilitated breeding strategies, such as marker-assisted selection (MAS), genomic selection (GS), and genetic transformations such as clustered regularly interspaced short palindromic repeats (CRISPR)-Cas techniques, largely accelerating the development of elite legume varieties with improved yield, nutritional value, stress resistance, and climate resilience [9].

Recently, the term ‘panomics’ emerges to refer to integration of data across various omics layers—genomics, transcriptomics, proteomics, metabolomics, and beyond—to provide a comprehensive view of the complete information flow within a plant [10]. This holistic approach enables a deeper understanding of how genetic and environmental factors interact to shape plant traits and further facilitates mining of key genes useful for molecular breeding. This review aims to summarize the latest advances in the world’s major legume crop improvement, focusing on the area of panomics. As advances in soybean omics, functional genomics, and molecular breeding has been recently reviewed [11]. Six food legumes are included hereby: three warm-season species (common bean, mungbean, and cowpea) and three cool-season species (pea, chickpea, and faba bean).

## Germplasm conservation status for major legume crops

Food legumes are often considered to have a narrow genetic base [12]. Notably, significant genetic loss has occurred, particularly during domestication and breeding for elite cultivars, with a reported 81% loss of total alleles and 23% loss of rare alleles in modern legume varieties [13]. To counter this, efforts to explore and conserve the genetic diversity of wild legumes have been underway since the mid-20th century. Today, more than 17 000 institutions worldwide house legume germplasm collections [14], reflecting growing recognition of the importance of wild species for breeding programs [15].

### Common bean

Common bean is arguably the most important grain legume for human consumption. According to Genesys Plant Genetic Resources (https://www.genesys-pgr.org/), a global portal to information about crop diversity conserved in genebanks, the International Center for Tropical Agriculture (CIAT, Palmira, Colombia) is the world’s largest custody for common bean germplasm, maintaining ~32 000 common bean accessions. With over 17 000 accessions, the National Rice and Beans Research Center (CNPAF, Brasilia, Brazil) of the Empresa Brasileira de Pesquisa Agropecuária, or EMBRAPA, is the second largest holding institute, followed by the National Center for Genetic Resources and Biotechnology (CENARGEN, Brasilia, Brazil), also under EMBRAPA, and the Western Regional Plant Introduction Station, USDA-ARS, Washington State University, both holding over 13 000 accessions, respectively. Genebanks in Germany, Russia, and Hungary also contain thousands of accessions (https://www.genesys-pgr.org/a/overview/v2EYGYrROyg, accessed Jan. 20, 2025). In Mexico, the National Institute for Forestry, Agriculture and Livestock Research (INIFAP) maintains over 8900 accessions for common bean (FAO-WIEWS 2022, https://www.fao.org/wiews, accessed Jan. 20, 2025). In Asia, the National Crop Germplasm Bank (Beijing, China), holds over 6500 accessions, including wild types, landraces, and cultivars (personal communication). Further, the National Gene Bank of India conserves more than 4000 accessions [16].

### Faba bean

Faba bean is a staple food in the Mediterranean region and across Eurasia. The wild faba bean is presumed to be extinct, meaning that all existing faba bean germplasm is available only from germplasm banks and locally grown cultivars [17]. As of 2008, 37 germplasm collections worldwide held 38 360 accessions [17]. Prior to its relocation to Lebanon in 2012, the International Center for Agricultural Research in the Dry Areas (ICARDA) genebank in Aleppo, Syria, was the largest repository, housing approximately 9000 accessions. The National Crop Genebank of China currently holds 4115 faba bean accessions (https://www.cgris.net/, accessed Jan. 20, 2025). The Svalbard Global Seed Vault (SGSV) of Norway preserves more than 6100 accessions deposited by 28 international depositors (https://seedvault.nordgen.org/, accessed January 20, 2025).

### Mungbean

Mungbean’s cultivation is concentrated in India and Southeast Asia. Mungbean germplasm is mainly conserved in institutions across Asia, including the Indian Council of Agricultural Research (ICAR)-National Bureau of Plant Genetic Resources (NBPGR) (~4500 accessions, http://genebank.nbpgr.ernet.in/), the Institute of Crop Sciences, of Chinese Academy of Agricultural Sciences (CAAS, >4120 accessions, https://www.cgris.net/home, accessed January 20, 2025), the World Vegetable Center (formerly the Asian Vegetable Research and Development Center, AVDRC) with >10 000 accessions (www.genebank.worldveg.org, accessed January 20, 2025), and the Plant Genetic Resources Conservation Unit of the University of Georgia, USA (>3000 accessions, https://npgsweb.ars-grin.gov/, accessed January 20, 2025). Other major collections are located in Russia, and Australia.

### Cowpea

As a crucial crop in sub-Saharan Africa, cowpea is well represented in the International Institute of Tropical Agriculture (IITA) genebank in Nigeria, which houses over 18 000 accessions. The Agricultural Research Service of the United States Department of Agriculture (USDA-ARS) also holds over 8000 cowpea accessions (https://www.genesys-pgr.org/c/cowpea, accessed January 20, 2025). In Brazil, regional germplasm centers such as Embrapa Recursos Genéticos e Biotecnologia (4928 samples) and Embrapa Meio Norte (3785 samples) are also notable reservoirs of genetic diversity (https://www.croptrust.org/knowledge-hub/crops-countries-and-genebanks/crops/cowpea/, accessed January 20, 2025). Additionally, the NBPGR of India holds over 4000 cowpea accessions (https://nbpgr.org.in/nbpgr2023/genebank-status-2/, accessed on January 20, 2025). In China, the CAAS maintains a total of 2818 accessions (https://www.cgris.net/, accessed January 20, 2025).

### Chickpea

Chickpea is one of the earliest domesticated crops and serves as a staple food in South Asia and East Africa. The largest collection of chickpeas and its wild *Cicer* relatives is maintained at the ICRISAT genebank, with over 20 504 accessions (https://genebank.icrisat.org, accessed Jan. 20, 2025). Other prominent collections include the NBPGR, India (>14 000 accessions) (https://nbpgr.org.in/nbpgr2023/genebank-status-2/, accessed January 20, 2025) and the ICARDA (~13 000 accessions) [18]. Genebanks in China, Russia, and France also contain rich germplasms [19].

### Pea

The pea originated in the Near East, particularly in modern-day Türkiye, Syria, and Jordan, around 9000 to 10 000 years ago. The National Institute for Agricultural Research (INRAE, France) is the world’s largest genebank for pea, with over 8800 pea accessions [20]. The Australian Grains Genebank, Agriculture Victoria (AGG) contains ~7400 accessions. The Western Regional Plant Introduction Station, USDA-ARS, Washington State University holds >6200 accessions. Other prominent holdings include the Genebank of Leibniz Institute of Plant Genetics and Crop Plant Research (IPK, ~5400), ICARDA, Lebanon (~4500), Germplasm Resources Unit, John Innes Centre, Norwich Research Park (~3500), and Science and Advice for Scottish Agriculture, Scottish Government (~3300) (https://www.genesys-pgr.org). Beyond this, the National Crop Germplasm Bank (Beijing, China, >7000 accessions), the NBPGR (New Delhi, India, >4700 accessions), the Nordic Genetic Resource Center (Alnarp, Sweden, >2000 accessions) also preserves significant amounts of pea germplasm.

## Recent advances in phenotyping technologies and phenomics in major food legumes

Precise phenotyping plays a crucial role in understanding how genetic and environmental factors influence plant physiology, growth, and development [21, 22]. Plant phenomics refers to the study of the plant phenome, which encompasses the complete set of observable characteristics and traits of a plant or plant tissue, all determined by the interaction between the plant's genome and its environment [23, 24]. Traditionally, phenotyping and the compilation of plant phenomes have been resource-intensive, requiring significant costs and labor [25]. However, recent technological advancements, represented by a range of high-throughput phenotyping (HTP) platforms, are revolutionizing this process. The integration of multi-dimensional devices, including unmanned aerial systems (UAS) or unmanned aerial vehicles (UAV), handheld and distributed phenotyping instruments, robotic systems, and lysimeter arrays, is transforming the study of plant phenomes [26]. Further, to accelerate data interpretation, machine learning algorithms and trait prediction models have been developed. These analytical tools significantly advance our understanding of genotype-to-phenotype relationships [27–30]. In this section, we review the most recent advances in phenotyping key traits in each crop (Table 1).

**Table 1:** Recent high-throughput phenotyping platforms applied in food legume crops.

**Type**	**Sensors**	**Traits Phenotyped**	**Species**	**Image**	**Reference**
Ground- and tower-based	3-D ChlF and MS imaging	nutrient deficiency	common bean	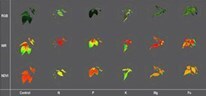	[31]
	HS; UAV with MS and thermal cameras	drought response; canopy volume, NDVI	common bean	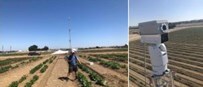	[32]
	conveyor belt, integrated with RGB, MS, HS camera	drought stress	common bean	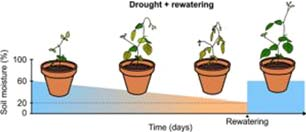	[33]
	NIRS	seed protein, oil and oleic acid content	faba bean	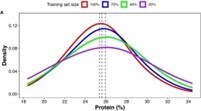	[34]
	Lemnatec platform, gravimetric, 3-D RGB, NIRS	growth rates and WUI	mungbean	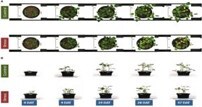	[35]
	LeasyScan with PlantEye^®^ sensors, 3D imaging and lysimeter	plant-water relation	cowpea	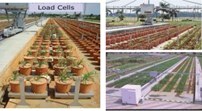	[36]
	PlantArray^®^, lysimeter	transpiration; soil water content	cowpea	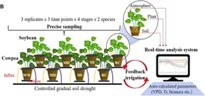	[37]
	Lemnatec platform, gravimetric, RGB, NIR, IR, and ChlF	drought-related traits	chickpea	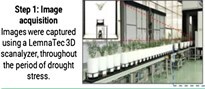	[38]
	Plant Accelerator^®^, RGB camera	salt tolerance	chickpea	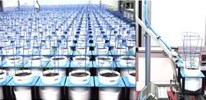	[39]
	PlantScreen^®^, ChlF	AGB, Photosystem II efficiency, under cold stress	pea	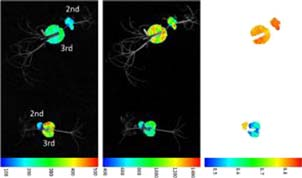	[40]
UAV/UAS integrated sensors	UAV, with RGB sensor	relative maturity, PH, stand count	common bean	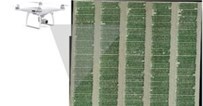	[41]
	UAV, with 2-D and 3-D RGB sensors	PH and yield	faba bean	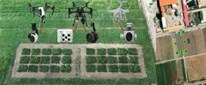	[42]
	UAV, with MS, RGB sensors	yield	faba bean	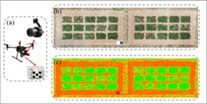	[43]
	UAV, with MS and RGB sensors	PH, SPAD, yield	faba bean	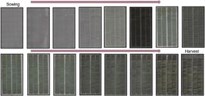	[44]
	UAV, with RGB, MS, TIR sensors	harvest index	pea, faba bean	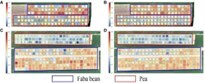	[45]

(Continued)

**Table 1:** Continued

**Type**	**Sensors**	**Traits Phenotyped**	**Species**	**Image**	**Reference**
	UAV, with RGB, MS, TIR sensors	AGB, yield	faba bean	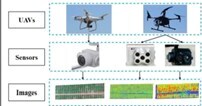	[46]
	UAV, with RGB and MS sensors	AGB, stomatal conductance, OSAVI, NDVI, NDRE	mungbean	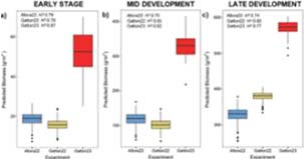	[47]
	UAV, with MS sensor	yield, dates to 50% flowering, days to physiological maturity	pea, chickpea	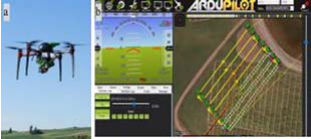	[48]

HS, hyperspectral sensor; MS, multispectral sensor; RGB, red-green-blue sensor; TIR, thermal infrared sensor; NIRS, near-infrared spectroscopy; ChlF, chlorophyll fluorescence; NDVI, normalized difference vegetation index; OSAVI, optimized soil-adjusted vegetation index; NDRE, normalized difference red edge; PH, plant height; AGB, above-ground biomass; WUI, water use index; 2-D, two-dimensional; 3-D, three-dimensional.

### Common bean

The common bean is primarily cultivated in regions with limited rainfall and irrigation, making drought resistance a critical objective in breeding programs. Several phenotyping platforms focusing on plant–water relations, leaf morphology, and physiological traits enable rapid screening of bean characteristics under genotype–environment interactions [49]. For monitoring common bean pod growth, nuclear magnetic resonance (NMR) sensor-based phenotyping tool has proved an effective HTP technology [50]. In 2016, an automated, image-based HTP platform was developed to assess root architecture in the field, supporting in-field identification and selection of bean and cowpea genotypes [27]. Subsequently, HTP platforms have been developed for the efficient evaluation of plant water status. For example, the PlantArray® system, which uses lysimetric measurements, allows real-time, high-throughput monitoring of water-use traits under controlled stress conditions, facilitating precise analysis of drought responses. By correlating physiological data with genetic markers, this platform aids in identifying key genetic determinants of drought resilience [21]. Lazarević *et al*. used chlorophyll fluorescence (ChlF) and multispectral (MS) traits to differentiate nutrient deficiency stress [31]. Hyperspectral remote sensing also offers a promising high-throughput approach for assessing drought tolerance in beans, enabling the rapid screening of physiological traits associated with drought response [32]. The developed ground-based partial least squares regression (PLSR) models have shown utility in predicting key indicators of drought response, such as stomatal conductance and predawn leaf water potential. Recently, Verheyen *et al*. developed a high-throughput phenotypic imaging system to evaluated the drought resistance of beans [33]. Drone-based phenotyping for common bean has also been reported, focusing on maturity [41].

### Faba bean

Recent advancements in drone and sensor technology have significantly expanded their application in faba bean phenomics. Ji *et al*. [42] employed two-dimensional (2D) and three-dimensional (3D) red-green-blue (RGB) color light sensors to create Crop Surface Models (CSM), using the maximum value of the CSM to represent plant height, achieving a coefficient of determination (R^2^) ranging from 0.93 to 0.99. They also estimated faba bean yield based on plant height using three machine learning algorithms, with the highest R^2^ value reaching 0.72. In 2023, they demonstrated the effectiveness of RGB sensors and ensemble learning (EL) models in extracting texture, structural information, and vegetation indices to estimate faba bean yield and biomass [51]. With the rise of sensor data fusion, Cui *et al*. estimated faba bean yield by combining RGB and MS sensor data with four machine learning algorithms, achieving an R^2^ value of 0.70 [43]. Additionally, the integration of UAVs equipped with RGB and MS cameras, along with machine learning algorithms, has proven accurate for predicting yield and chlorophyll content. The optimal growth stages for measuring SPAD (leaf chlorophyll content) and yield were identified as BBCH 50 (flower bud present) and BBCH 60 (first flower open), respectively [44]. Although limited research has focused on estimating the harvest index, Ji *et al*. used multi-source data fusion with RGB, MS, and thermal infrared (TIR) sensors and ensemble Bayesian model averaging in field experiments conducted over 2 years to estimate the harvest index of faba bean at multiple growth stages, achieving an accuracy of 0.64 [45]. More recently, Ji *et al*. enhanced the accuracy of faba bean biomass and yield estimation by employing the XGBoost algorithm and multisensor data fusion, achieving R^2^ values of 0.75 and 0.79, respectively [46].

Near-infrared spectroscopy (NIRS) data have also been widely used to predict seed quality attributes of faba beans, such as protein, starch, oleic acid, total polyphenols, and bioactive compounds, using various predictive models [34,52,53]. Given that models like support vector machines (SVMs) and neural networks, such as artificial neural networks (ANNs) and convolutional neural networks (CNNs), require extensive datasets for training, alternative models, including partial least squares (PLS), elastic net (EN), memory-based learning (MBL), and Bayes B (BB), were employed to predict seed quality in faba beans grown at various locations, with best prediction performance achieved for protein and oil content [34].

### Mungbean

Rane *et al*. employed a high-throughput phenomics system, supported by a high-resolution camera, to investigate 24 elite mungbean genotypes under controlled water stress over a 25-day period [35]. This study provided valuable insights into the growth and production patterns of mungbeans under varying soil moisture conditions. Chiteri *et al*. conducted image-based analysis of leaf traits in 484 mungbean accessions. The extracted morphological parameters were used for association mapping, successfully identifying candidate genes associated with leaf length, width, perimeter, and area [54]. Additionally, an interaction regression model, incorporating leaf length and width as predictors, was developed to estimate ovate leaflet area, achieving an adjusted R^2^ of 0.97. As a nondestructive, image-based phenotyping tool, this platform is expected to be valuable for future studies on canopy dynamics under various stress conditions. Recently, UAV-based MS and RGB sensors were applied for phenotyping and predicting mungbean agronomic and physiological traits, including early vigor, aboveground biomass and stomatal conductance [47].

### Cowpea

Canopy traits, such as leaf area, leaf area index, and transpiration, are essential for understanding plant-water relations. Vadez *et al*. developed a non-destructive, image-based platform (LeasyScan) combined with lysimetric capacity to assess canopy traits in plants, including cowpea [36]. A strong regression coefficient (0.93) was observed between leaf area measurements and data derived from LeasyScan analysis for cowpea. Another HTP, PlantArray®, evaluated the whole-plant water relations of 106 cowpea accessions by integrating a gravimetric system, atmospheric and soil probes, irrigation valves, and a controller. The resulting phenotypic data were used in a genome-wide association study (GWAS), identifying 20 SNPs associated with stomata-mediated drought-responsive traits [55]. Further integration of this system with transcriptome analysis revealed that the *VuHAI3* and *VuTIP2;3* genes may play roles in the dehydration avoidance mechanism of cowpea [37]. Yu *et al*. developed a lower-cost, image-, and weight-based system to monitor shoot growth and evapotranspiration in a cowpea diversity panel. This platform was validated by integrating phenotypic data with GWAS, identifying nine genetic loci potentially associated with drought tolerance [56].

### Chickpea

HTP has been applied to study plant traits in chickpea under salinity, drought, and temperature stresses [38,57–59]. A comprehensive HTP study of 60 chickpea accessions under contrasting water regimes demonstrated that color-related traits were effective indicators of stress progression [57]. ChlF imaging has provided valuable insights into photosynthetic efficiency under water deficit conditions and the early detection of Fusarium wilt in chickpea [38,58]. The Lemnatec platform, which integrates nondestructive imaging technologies, such as RGB, NIR, IR, and ChlF, has been crucial in phenotyping drought-related traits in chickpea, including stomatal conductance and photosynthetic activity, achieving strong correlations between manually recorded and image-based traits [38]. Phenotyping platforms for yield and phenology of chickpea and pea have also been reported, using UAV integrated with MS cameras [48]. An HTP platform, the Plant Accelerator^®^ has been developed for evaluating salinity tolerance in chickpea [39].

To accelerate the assessment of energy dissipation, theoretical nonphotochemical quenching (NPQ(T)) was introduced for high-throughput phenotyping. NPQ(T) evaluations revealed that desi chickpeas maintained higher Estimated Biovolume under drought conditions compared to kabuli types, a result attributed to more efficient energy dissipation in photosystem II [59]. Additionally, an efficient, image-based method for measuring chickpea seed size was developed by Sankaran *et al*., achieving a high correlation coefficient of 0.90 [60].

### Pea

Imaging sensors, when combined with advanced processing techniques, have shown significant potential for monitoring pea flowering intensity. Strong correlation coefficients were observed between UAV integrated and proximal RGB image data and visual rating scores, demonstrating the effectiveness of these technologies for assessing flowering [61]. UAV-based MS imaging systems have also been employed for high-throughput phenotyping to predict seed yield and maturity in peas. For example, Zhang *et al*. [48] utilized a quadcopter UAV equipped with a five-band MS camera to capture spectral images of dry pea across three growing seasons and three locations. They identified 3 to 20 image-based features, and the combined feature dataset, analyzed using LASSO regression, achieved an R^2^ value of 0.80 in pea. In a similar study, Bazrafkan *et al*. [62] assessed the effectiveness of five machine learning algorithms, coupled with UAV-based light detection and ranging (LiDAR) and MS data, for predicting dry pea maturity. They found that incorporating multi-sensory data yielded the most accurate predictions. Recently, Liu *et al*. collected dual-sensor data (RGB + MS) from UAVs to estimate pea yield using EL and four base learners. They found that estimation accuracy can be optimized by using fusion data obtained at the mid-filling stage for estimation. The EL algorithm achieved the best performance than base learners [63].

A high-throughput phenotyping platform, Plant Phenomics Victoria, has proven useful in predicting early vigor traits in field pea. Strong correlations were found between estimated parameters from the platform and manual measurements [64]. Furthermore, cold tolerance in field pea has been evaluated using the image-based, automated high-throughput platform PlantScreen®. With a throughput of 16 plants per hour, this platform allows for the simultaneous, automated analysis of shoot biomass and photosystem II efficiency via ChlF imaging, aiding research into cold tolerance mechanisms in pea [40].

## Omics resources for major food crops

**Figure 2:**
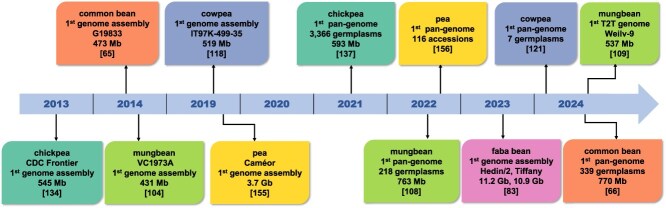
Key milestone events in genomic advances of food legumes. The first reference genome assembly, first pan-genome and telomere-to-telomere (T2T) genome assembly are presented. For the first genome assemblies and T2T genome, the cultivar, and genome size are presented in each box. For the first pan-genome, the number of accessions/germplasms is presented in the box.

As of now, at least 143 reference and 72 annotated genomes of Fabaceae plants have been assembled (https://www.ncbi.nlm.nih.gov/datasets/genome/?taxon=3803, accessed January 20, 2025). Among these, several milestone progresses have been made in the genomics of six food legume crops (Fig. 2). Beyond genomics, substantial progress has also been made in other omics fields, including transcriptomics, proteomics, metabolomics, and epigenomics. Collectively, these omics approaches provide a comprehensive view of legume biology, from genetic information to functional outcomes. To deposit these resources, both general and legume-specific databases and web tools have been available (Table 2). In addition to omics data, these databases and web portals also encompass rich information of genetic maps, germplasm, quantitative trait loci (QTL), and others. The following sections detail the multi-omics resources available for each legume crop.

**Table 2:** Genomics, proteomics and metabolomics databases for major legume species.

**Omics**	**Database**	**Description**	**Species** ^**a**^	**Launch year**
Genomics	LegumeIP	integrative database for comparative genomics and transcriptomics of model legumes/genomics and RNA-seq-based transcriptomics data	22	2012
	Pulse Crop Database (PCD), formerly Cool Season Food Legume Genome database (CSFL)	genomes, genes, transcripts, genetic maps, markers, QTL, germplasm, genomic, genetic, and breeding resources phenotype publications, with integrated tools	15	2014
	Vigna Genome Server	genome assembly and annotation data	13	2016
	KnowPulse	germplasm; genomic data; phenotypic data	14	2019
	SoyBase	a USDA genetic and genomics database contains soybean genetic and genomic data	1	Early 1990s
	*Vicia faba* Omics database (*Vf*ODB)	a web portal of faba bean germplasm information, ESTs, EST-SSRs, and mitochondrial-simple sequence repeats (MtSSRs), microrna-target markers, and genetic maps	1	2020
	Cowpea Genespace/Genomics Knowledge Base (CGKB)	cowpea annotation knowledge base, based on 298 848 cowpea genespace sequences (GSS) isolated by methylation filtering of genomic DNA	1	2007
	Chickpea Genomic Web Resource (CGWR)	web tools for chickpea genome visualization and comparative analysis	1	2014
	*Cicer* Methylation Variation Map (*Cicer* MethVarMap)	a web-based resource focused on DNA methylation variation in chickpea	1	2024
Proteomics	ProMEX	mass spectral reference database, tryptic peptide fragmentation mass spectra data derived from plants	5	2012
	LegProt	legume-specific protein database consisting of amino acid sequences	7	2011
	Medicago PhosphoProtein Database	Medicago phosphorylation data	1	2012
	Soybean Proteome Database	reference maps of soybean (*Glycine max* cv. Enrei) proteins collected from several organs, tissues, and organelles, under flooding, salt, and drought stress	1	2009
Metabolomics	Soybean Knowledge Base (SoyKB)	integrative information on soybean genomics, transcriptomics, proteomics, and metabolomics	1	2014
	MedicCyc	a *M. truncatula*-specific pathways database containing over 250 pathways with related genes, enzymes, and metabolites	1	2007

a The total number of legume species included in the database.

### Common bean

The first reference genome of the common bean was established in 2014, sequencing 473 Mb of the 587-Mb genome from the Andean landrace G19833 [65]. More recently, Cortinovis *et al*. constructed the first *Phaseolus vulgaris* pan-genome, based on five *de novo* genome assemblies from wild and domesticated genotypes, alongside short-read whole-genome sequencing (WGS) data from 339 common bean accessions [66].

In addition to genomic studies, high-throughput RNA sequencing (RNA-seq) has been pivotal in identifying the transcriptional networks underlying stress tolerance mechanisms in beans. These studies have revealed that genes related to water stress, phosphorus use, heavy metal, and disease resistance are differentially regulated in roots and aboveground tissues [37,67–70]. Proteomics studies in common bean have identified differentially expressed proteins (DEPs) linked to traits associated with terminal drought stress [71], biological nitrogen fixation [72], low water potential stress [73], and resistance to halo blight [74]. Two major common bean protein databases, ProMEX and LegProt, are publicly available (Table 2).

Since the completion of the first reference histone–DNA interaction map [75] and nucleotide-resolution methylomes through whole-genome bisulfite sequencing [76], many studies have highlighted the critical regulatory role of epigenetic modifications in the common bean regulome. The roles of DNA methylation, histone modification, and small RNAs in the symbiotic process have been reviewed or demonstrated to be associated with root nodule formation, pod string development, disease resistance genes, and abiotic stresses [77–80]. In 2021, a high-resolution (~2 kb) Hi-C chromatin architecture map for common bean was generated [81]. A small RNA sequencing identified a virus-derived small interfering RNA as a trans-kingdom epigenetic regulator conferring drought tolerance [82].

### Faba bean

The 13-Gb faba bean genome (2n = 2x = 12) is one of the largest diploid field crops. In 2023, a chromosome-scale assembly of the faba bean genome was made publicly available [83]. A high-quality faba bean reference transcriptome, constructed from 37 samples, enabled the identification and correction of 121 606 transcripts, as well as the prediction of alternative splicing events, long noncoding RNAs (lncRNAs), and fusion transcripts [84]. Single-nucleotide polymorphism (SNP) genotyping platforms based on next-generation sequencing (NGS) have expanded the available genomic tools for targeted breeding [85, 86], generating high-density SNP markers from various international panels [87].

RNA sequencing (RAN-Seq) has been widely applied for exploring genetic diversity of faba beans, leading to *de novo* transcriptome assemblies of faba bean [88–91] responsive to water stress [92], cold stress [93], and Ascochyta fabae infection [90]. The discovery of a large number of transcripts and high-quality unigenes from seed, leaf, seedling, and root tissues has enriched the genomic resources available to faba bean breeders [94, 95]. More recently, single-molecule, real-time (SMRT) full-length transcriptome sequencing on the PacBio Sequel platform has been used to identify a high abundance of vernalization-related transcripts and flowering-related genes [96]. As this platform is appropriate for long-read sequencing, a high functional annotation ratio of 95.5% was obtained. RNA-Seq of the faba bean seeds also discovered significant pathways related to seed hydration capacity and seeds staining traits related to the Pea seed-borne mosaic virus (PSbMV) [97].

Metabolomics studies in faba bean have focused on quality and nutritional value [98], bitter perception [99, 100], polyphenols and flavonoids in faba bean flowers [101], and resistance to Fusarium wilt [102]. To highlight potential bitter nonvolatile compounds in *V. faba*, ultra-high-performance liquid chromatography with a diode array detector and tandem high-resolution mass spectrometry (UHPLC-DAD-HRMS), along with bitter perception testing, was used to identify forty-two tentatively nonvolatile compounds [99].

### Mungbean

To date, over 10 mungbean (493.6–579.0 Mb, 2n = 2x = 22) accessions, including cultivars, wild relatives, and polyploid species, have been subjected to WGS [103–105]. At least five sets of mungbean genomic data are publicly available: VC1973A, IPU-02-03, VR01, RIL59, and Kamphaeng Saen 1. The first draft of the mungbean genome, based on the cultivar VC1973A, was completed in 2014, resulting in 2748 scaffolds on a 431-Mb map [104]. In 2021, the genome assembly was improved, expanding to 476 Mb with a much higher N50 of 5.2 Mb [106]. Other high-quality reference genomes have since been released, including that of the Chinese cultivar ‘Sulv1’, assembled using nanopore long reads, Illumina short reads, and Hi-C data [107]. In 2022, the first mungbean pan-genome was constructed [108]. In 2023, a gap-free, telomere-to-telomere (T2T) assembly for the mungbean cultivar ‘Weilv-9’ was completed [109].

Transcriptome analysis has been used to develop EST-SSR markers in mungbean for novel gene discovery and marker-assisted breeding [110]. Transcriptomes from four different tissues (leaf, flower, root, and pod) were sequenced for VC1973A, and the transcriptome sequences of 22 *Vigna* accessions from 18 species were utilized for genome annotation [104]. The genome annotation of ‘Weilv-9’ incorporated 214 NGS transcriptomic datasets to improve its robustness [109]. Transcriptomic resources also focus on biotic and abiotic stress responses in mungbean, such as responses to mungbean yellow mosaic virus (MYMV) [111] and drought [112]. Mungbean proteomic and metabolomic resources are available for studying seed development and germination, using techniques like 2-DE, nanoelectrospray mass spectrometry, and NMR [113, 114]. Wu *et al*. discovered that 63 metabolites dominated the mungbean seed metabolome, including lipids, amino acids, oligo−/monosaccharides, cyclitols, cholines, organic acids, nucleotides/−sides, nicotinates, and secondary metabolites from the shikimate pathway [114].

The whole-genome methylation profile of mungbean was obtained using two cultivars, VC1973A and V2984 [115]. The results indicated that DNA methylation regulates the expression of paralogous genes in VC1973A. Later, the epigenetic regulation of synchronous pod maturity (SPM) in mungbean was investigated by DNA methylation profiling of eight recombinant inbred lines and their parental genotypes [116]. Furthermore, hypermethylation of differentially methylated regions (DMRs) may contribute to stress resistance, such as drought tolerance [117].

### Cowpea

The first fully assembled cowpea (640 MB, 2n = 2x = 22) genome was completed in 2019 for the grain cowpea line IT97K-499-35, with a genome size of 640.6 Mb [118]. Since then, sequencing efforts have accelerated, and genomes from 13 independent cowpea lines have been assembled. Subsequent assemblies, such as that of the vegetable cowpea line Xiabao II (632.8 Mb), have provided further insights into breeding strategies [119]. In 2023, a more accurate genome assembly for Xiabao II was completed using HiFi long-read sequencing and Hi-C technology, covering 594 Mb [120]. A comprehensive cowpea pan-genome, incorporating multiple germplasm resources, has also been constructed, shedding light on genes related to stress responses and agronomic traits [121]. In 2024, an analysis of 344 germplasm resources led to the discovery of genomic regions associated with agronomic traits such as pod length and stress resistance, providing valuable information for future breeding programs [122].

Transcriptome analysis, alongside small RNA sequencing, has improved our understanding of the differentially expressed mRNAs and miRNAs related to response to *Rhizosphere Priestia,* drought, salt, virus, and aphid in cowpea [123–127]. Transcriptome and metabolome integration has been used to analyze the regulatory mechanisms of cowpea senescence induced by exogenous MEL and nitric oxide [128, 129], as well as metabolic disruptions caused by pesticides [130]. Metabolomic technologies have further enhanced the identification of trait regulation in cowpea. For example, metabolite profiling has detected 34 secondary metabolites in the cowpea seed coat, including phenolic acids, flavonoids, anthocyanins, sphingolipids, and fatty acids [131]. Accumulation of metabolites involved in flavonoid biosynthesis and the alpha-linolenic acid metabolism pathway has been suggested to contribute to resistance against *Megalurothrips usitatus* in cowpea [132]. Recently, a multi-omics comprehensive analysis was conducted in cowpea to investigate the effect and regulatory mechanisms of pre-storage low temperature on storage quality. This analysis identified several interesting differentially expressed genes (DEGs) belonging to the AP2/ERF, MYB, NAC, WRKY, and LOB transcription factor families [133].

### Chickpea

Chickpea (544 Mb, 2n = 2x = 16) genomics has made significant advances. Since the first genome assembly constructed in 2013 [134], there are at least five available genome assemblies and large-scale resequencing data. These efforts have enabled the development of over 2000 SSR markers, a 15 000-feature DArT platform, and millions of SNP markers for chickpea improvement, with the Axiom® CicerSNP Array now being widely used for trait mapping [135, 136]. Whole-genome resequencing (WGRS) of parental lines and cultivars has provided insights into genetic diversity, high-density trait mapping, and the identification of key candidate genes for agronomic traits, as well as the study of chickpea's origin and migration [9]. Mapping variation in 3171 cultivated and 195 wild chickpea accessions helped construct a pan-genome that captured the genomic diversity in cultivated and wild progenitors. This analysis identified chromosomal segments and genes under selection during domestication, migration, and improvement, and pinpointed deleterious mutations affecting fitness in elite chickpea germplasm [137]. Building on this, Khan *et al*. developed a super-pangenome based on eight wild *Cicer* species, identifying 24 827 gene families—14 748 core, 2958 softcore, 6212 dispensable, and 909 species-specific [138].

Extensive transcriptomic data are available on chickpea’s drought responses [139]. Basso *et al*. identified thousands of differentially expressed transcripts and ten candidate genes that regulate branching in chickpea. Proteomics studies in chickpea have mainly focused on drought stress responses [140]. Several studies have highlighted the role of ribulose-1,5-bisphosphate carboxylase/oxygenase (RuBisCO), ATP synthase, and l-ascorbate peroxidase in drought resistance [141, 142]. A nontargeted analysis of 36 chickpea genotypes under drought stress identified key metabolites, including l-threonic acid, fructose, and sugar alcohols, associated with drought adaptation [143]. Another study highlighted resistance-associated metabolites, such as ferulic acid in moderately resistant chickpeas, while catechins, phthalic acid, and nicotinic acid were more abundant in susceptible varieties. Additionally, infected susceptible cultivars showed increased salicylic acid (SA) levels and suppressed MeJA, revealing the role of phytohormones in chickpea–*Ascochyta rabiei* interactions [144]. Recently, multi-omics strategy was employed to identify key proteins involved in antibiotic biosynthesis, galactose metabolism, isoflavonoid biosynthesis, and drought-response mechanisms [145, 146].

DNA methylation diversity in chickpea surpasses genetic diversity, driving phenotypic variation and supporting the crop’s evolution and domestication. The *Cicer MethVarMap* database (http://223.31.159.7/cicer/public/) offers valuable tools for crop improvement, emphasizing the role of epigenetics in enhancing chickpea’s diversity despite its narrow genetic base [147]. Chickpea drought-sensitivity may be attributed to hypomethylated ribosomal genes and impaired ribosomal biosynthesis, as revealed by whole-genome bisulfite sequencing (WGBS). Small RNA sequencing has proven a robust tool for mining novel chickpea miRNAs and their respective target genes associated with seed development [148], responses to heavy metal exposure [149], and resistance to *Fusarium* wilt infection [150]. Several miRNAs, such as miR172c, miR394, and miR1509, have been validated for their positive role in increasing root nodule number [151].

### Pea

Pea (3.7 Gb, 2n = 2x = 14) genomics has advanced more slowly compared to other major legumes, mainly due to its large and complex genome [152, 153]. Before the pea genome sequence was available, microarray-based transcriptome-wide analysis was a widely used tool for studying molecular basis of developmental stages and stress responses in pea [154]. The first reference genome of pea (*cv*. Caméor) was published in 2019 [155]. In 2022, the CAAS released an improved reference genome for the cultivar ZW6, with a 243-fold increase in contig length and improved continuity and sequence quality compared to the previous assembly [156]. Additionally, the study constructed a pan-genome using 116 cultivated and wild pea varieties. In 2024, a high-quality reference genome for the pea cultivar *Zhewan No.1* was released, along with a genetic variation map based on 314 accessions [157]. These advancements have allowed for the rediscovery of the genetic basis of Mendelian traits and other key agronomic traits in pea.

Two-dimensional electrophoresis (2-DE), matrix-assisted laser desorption ionization-tandem-time-of-flight (MALDI-TOF/TOF) tandem mass spectrometry, and liquid chromatography-mass spectrometry (LC–MS)-based proteomics have been widely used to analyze the pea proteome in response to biotic and abiotic stresses [153, 158] The generation of a pea embryo proteome map enabled comprehensive annotation of the functions and intracellular localization of pea seed proteins [159]. Recently, label-free quantitative proteomics was used to analyze round and wrinkled pea seeds at different stages, revealing key differences in protein profiles and starch metabolism between these two seed types [160].

## Panomics-empowered discovery of breeding target genes

**Figure 3:**
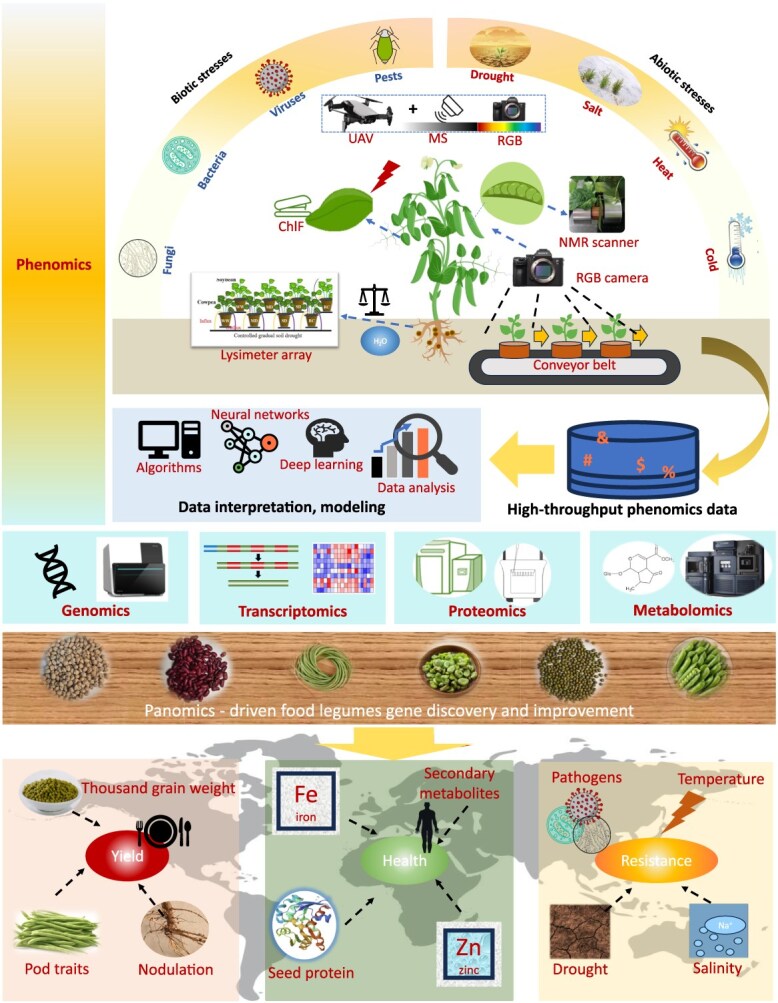
A modern working flow using panomics data to empower food legumes improvement. The increasing availability of various high-throughput phenotyping platforms, which rely on different sensors, has greatly enhanced the study of food legume phenomics. This advancement, when combined with other omics technologies, forms a panomics-driven framework for improving food legumes. The image for NMR scanner is reproduced with premission from [50].

Over the past decades, the major target of breeding in the food legumes have been increase of yield, improvement of nutrient value, and enhanced stress resilience in the context of climate change. This section summarizes the key discoveries in gene mining empowered by panomics and the practice of breeding in these fields (Fig. 3).

### Yield and quality

#### Common bean

In common bean, two anti-yield genes on chromosome Pv09, *Phvul.009G190100* and *Phvul.009G202100*, were identified through comparative genomic analysis. These genes, encoding basic leucine zipper (bZIP) transcription factors, showed a negative correlation with seed yield [161]. The *Phvul.006G072800*, encoding the β-1,3-glucanase 9 protein, was determined as the causal gene for *PvPW1* underlying pod width [162]. To address the drawback of low levels of the essential amino acid methionine in this crop, the *be2s1* gene, which codes for a methionine-rich storage albumin from Brazil nuts, was introduced via biolistic methods, resulting in a 14–23% increase in methionine content in transgenic lines [163]. *PvZFL1*, *PvZFL10*, and *PvNRAMP9* controls seed zinc content [164]. Three galactinol- and two RFO-synthase genes have been characterized for tissue-specific expression; these candidate genes may play a pivotal role in reducing the RFO content in bean seeds [165]. Recently, the metal tolerance protein (MTP) gene family of the common bean was identified via genome-wide analysis, which contains nine *PvMTP* genes located on 7 of 9 chromosomes. The effect of Fe and Zn treatments on *PvMTP* genes expression was also investigated, demonstrating the potential of these genes for biofortification in legumes [166]. The *MTP* gene family, including cation diffusion facilitators, has been implicated in Fe and Zn homeostasis in plants like rice [167]. Additionally, candidate genes involved in specialized metabolite biosynthesis have been identified [168].

#### Faba bean

Faba bean has a large genome (~13 Gb), which has made genetic and gene mapping studies challenging. In 2023, however, high-density SNP genetic linkage maps were used to identify hundreds of QTLs for agronomic traits related to flowers, pods, plant types, and seed traits [169]. GWAS using different diversity panels of faba bean accessions identified rich markers associated with traits, including seed quality and yield-related traits [170, 171]. For bioactive compounds, an insertion mutation in the *VC1* gene is believed to be responsible for low levels of vicine and convicine in certain faba bean varieties [94]. The zero-tannin (zt) trait, which is controlled independently by two complementary recessive genes, *zt1* and *zt2*, has been linked to improved nutritional qualities. Webb *et al*. [172] and Gutierrez and Torres [173] reported that the *zt1* phenotype is encoded by the *TTG1* gene, an ortholog of the *Medicago* WD40 transcription factor. In faba bean, the zero-tannin trait is also associated with a white flower phenotype in lines carrying the *zt2* gene. The locus for zero-tannin content has been pinpointed to the *VfTT8* gene, a bHLH transcription factor [174].

#### Mungbean

Recently, a GWAS using 196 mungbean accessions identified *VrEmp24/25* and *VrKIX8* as candidate genes for seed length and 100-seed weight [175]. Other candidate genes include a leucine-rich repeat serine/threonine/tyrosine kinase (*Vradi05g00200*) for SP, a Calvin cycle protein (*Vradi03g06500*) for DFT, and a serine carboxypeptidase (*Vradi04g07810*) for pleiotropic traits (SPC, SSC, and HSW). [176]. Research on quality traits, such as phytic acid, seed starch, anthocyanins, and polyphenolic compounds, remains limited. *VrMYB90*, a member of the R2R3-type MYB family, was identified as a key regulator of anthocyanin biosynthesis [177]; this gene was also linked to the seed coat color trait [178, 179]. Polyphenolic compounds, specifically vitexin and isovitexin, are abundant in mungbean. Two genes (*jg15859* and *jg15860*) encoding glutamine synthetase may serve as a substrate for vitexin/isovitexin synthesis. Additionally, a SWEET10 homolog (*jg24043*) was associated with crude starch content [108].

#### Cowpea

Several QTLs associated with pod characteristics such as pod length, width, and chemical components have been identified [120, 180, 181]. In 2019, a combination of GWAS meta-analysis with synteny comparison in common bean identified six candidate genes (*Vigun05g036000*, *Vigun05g039600*, *Vigun05g204200*, *Vigun08g217000*, *Vigun11g187000*, and *Vigun11g191300*) controlling seed size [182]. Yang *et al*. combined genome, transcriptome, and metabolome analyses to identify *VuMYB114*, a transcription factor associated with pod color. In the WSS1 (green pod) line, a premature stop codon in *VuMYB114* prevents the activation of the anthocyanin biosynthesis pathway, leading to green pods rather than purple [183]. A multiomic data analysis highlighted several genes that coordinately control anthocyanin and flavonoid accumulation, including *VuMYB90-1*, *VuMYB90-2*, *VuMYB90-3*, *VuCPC*, *VuMYB4*, and endogenous bHLH and WD40 proteins [184].

#### Chickpea

Quality traits of chickpea have been investigated in many GWASs focusing on Fe and Zn, grain protein, sugar metabolism, grain fatty acids, starch, fiber [185, 186]. The gene *ROP1 ENHANCER1* was shown to play a crucial role in SPC determination through a knock-down experiment [187]. The linkage group *CaLG04 was found to co-localize with QTLs for* seed iron (Fe) and zinc (Zn), suggesting it could potentially enhance both nutrients [188]. A gene correlation network based on comparative transcriptome and metabolome analysis discovered several putative candidate genes like *CIPK25*, *CKX3*, *WRKY50*, *NAC29*, *MYB4*, and *PAP18* underlying Fe tolerance in chickpea [189]. The chickpea nicotianamine synthase 2 (*CaNAS2*) was suggested as a housekeeping role in systemic translocation of Fe. Overexpression of *CaNAS2* in chickpea seeds showed nearly doubled nicotianamine level, which might translate into increased Fe bioavailability [190].

#### Pea

QTLs underlying pea quality traits mainly seed starch content, seed mineral concentrations and contents have been studied using RIL population or natural diversity panels [191–193]. Several genes have been reported to regulate seed quality in pea. For example, the genes *R*, *Rb*, *Rug3*, *Rug5*, and *TAR2* regulates seed starch synthesis; while *Abi5*, *Lox-2*, and *Vc-2* controls protein synthesis [194]. A naturally occurring insertion mutation in the *SbeI* gene at the r locus has been demonstrated to be causative to increased resistant starch and the wrinkled-seeded phenotype, by reducing amylopectin synthesis. Genetic markers for the *SbeI* allele have been developed [195]. Recently, two comprehensive genetic studies on pea were conducted in 2022 and 2024. In 2022, Yang *et al*. analyzed the genetic basis of 12 agronomic traits using genotyping-by-sequencing, identifying 25 QTLs associated with important traits [156]. Later, in 2024, Liu *et al*. resequenced 314 pea accessions for a GWAS, identifying 235 candidate loci linked to 57 key agronomic traits, as well as candidate genes for known Mendelian traits [157]. Three loci, TI1, TI2, and Tri have been identified to encode three distinct trypsin inhibitors, thereby promoting nutrient adsorption [196]. A gene controlling pod softness, *PsPS1*, was fine-mapped to a ~ 6 Mb genomic region in pea, identifying *Psat1g150000* encoding a pectate lyase superfamily protein as a candidate gene [197].

### Abiotic stress tolerance

#### Common bean

Numerous studies have identified SNPs and QTLs associated with drought-related traits, such as osmotic protector biosynthesis [198], bioclimatic-based drought indices [199], revealing widespread drought adaptation genes on all chromosomes. RNA-Seq revealed repression of ABA-responsive genes *PP2C* (*Phvul.001G021200*) and a putative *ABA 8′-hydroxylase gene* (*Phvul.002G122200*) under drought conditions [200]. Notably, the aquaporin gene *PvXIP1;2* confers drought resistance at the seedling stage [201]. The *PvLTP* family genes may also contribute drought tolerance, with 9 *PvLTP* genes up-regulated under drought treatment [202].

Hiz *et al*. [203] identified a putative chalcone O-methyltransferase gene (*pvChOMT*) with a nearly fourfold upregulation upon salt stress. Ecotopic overexpression of *pvChOMT* in Arabidopsis suggested that *pvChOMT* can be a reliable candidate gene for breeding salt stress tolerance [204]. QTLs associated with reproductive traits, such as pollen viability and pod production under heat stress have been identified on chromosomes Pv05 and Pv08, alongside loci linked to flowering time and photoperiod sensitivity [205, 206]. Introgressions from *Phaseolus acutifolius* have also revealed QTLs that enhanced seed yield and reduced pod abortion under heat stress [207, 208].

#### Faba bean

Drought-responsive genes in the drought-tolerant faba bean variety Hassawi 2 have been identified [209]. Overexpression of a bZIP transcription factor *VfbZIP5* enhanced drought tolerance, possibly related to lower levels of proline (PRO), malondialdehyde (MDA), and peroxidase (POD) [210]. Potential genes underlying faba bean response to temperature have been studied using multi-omics approach. Faba bean vernalization were associated with 91 DEPs associated with photosynthesis and phytic acid metabolism, and a family of proteins, glycine-rich RNA-binding factor, as involved in alternative splicing on transcript abundance [211]. transcription factors helix–loop–helix bHLH143-like S-adenosylmethionine carrier, putative pentatricopeptide repeat-containing protein At5g08310, protein NLP8-like, and photosystem II reaction center PSB28 proteins may serve as potential genes underlying heat tolerance [212].

#### Mungbean

Mungbean is highly sensitive to abiotic stresses, particularly drought, salinity, and heat. Drought tolerance in mungbean has been studied extensively. Chang *et al*. identified transcription factors such as TCP, NAC, bZIP, and bHLH, as well as several protein kinase genes as candidate genes for drought tolerance [213]. A recent study fine-mapped *LOC106764181*(*VrYSL3*)**,** which encodes a yellow stripe1-like-3 protein to confer resistance to calcareous soil in mungbean [214].

For salt stress, a study identified 21 candidate genes, including *VrFRO8*, *VrNAS1*, *VrFTRB*, and *VrMAR1*, which cooperate to facilitate iron ion transport and reduce SOD contents under salt stress [215]. Other salt-tolerance genes include AMT (*Vradi07g01630*), OsGrx_S16-glutaredoxin (*Vradi09g09510*), and a dnaJ domain protein (*Vradi09g09600*) [216]. More recent studies have revealed the involvement of *VrWRKYs*, *VrPHDs*, and *VrMYBs* in salt stress response. Genes such as *VrPHD14*, *VrMYB96*, *VrWRKY49*, and *VrWRKY38* have been found to be significantly activated under salt stress [217]. Furthermore, members of the YUCCA family, the TATA-box binding protein (TBP)**,** and various TBP-associated factors (TAFs) have been shown to respond to multiple abiotic stresses, including salinity [218, 219].

#### Cowpea

A cowpea specific gene, *UP12_8740*, was shown to play a critical role in drought tolerance, as confirmed through expression analysis and functional validation [220]. Another gene *VuDREB2A* was isolated and characterized from cowpea; the gene has the ability to bind dehydration-responsive elements *in vitro* and confer enhanced drought resistance in transgenic Arabidopsis [221]. Salt stress during various stages of cowpea were studied, revealing numerous QTLs [222–224]. Transcriptomic studies on cowpea also identified salt-responsive DEGs, including several potential transcription factors [225]. NAC transcription factors, such as *VuNAC1* and *VuNAC2*, have been shown to enhance both drought and salt tolerance when overexpressed [226]. The Class-I *VuTCP9* in cowpea increases drought and salinity tolerance without altering water use efficiency (WUE) [227]. The gene also interacts with genes related to hormonal biosynthesis, stomatal development and abiotic stress responsiveness. *VunMED2* positively responds to cold stress of asparagus beans (*Vigna unguiculata* subsp. *sesquipedalis*), manifesting higher survival rate, ROS scavenging activity. This gene works synergistically with *VunHY5* to activate the expression of *VunERD14* [228]. Gene expression analysis of *VunMED* genes also implicated their potential role in cowpea during cold stress [229].

#### Chickpea

A genotyping-by-sequencing (GBS)-based analysis identified nine QTLs for drought-related traits, including the membrane stability index and yield, with a QTL on LG7 explaining more than 90% of the phenotypic variance for membrane stability [230]. Haplotype analysis confirmed five key QTLs, including qYLD7.1, which is linked to secondary metabolite biosynthesis. Candidate gene analysis identified 99 drought-responsive genes, offering potential targets for breeding. There are also some association studies for drought tolerance, uncovering candidate genes *FRIGIDA* and *CaTIFY4b* [231], *CPN60-2* and *hsp70* [232]. Gene annotation from a multi-trait GWAS found the EMB8-like and Ribosomal Protein Large P0 (RPLP0) protein may control salinity tolerance [233].

Heat stress, particularly when temperature exceeds 35°C, can reduce chickpea yields by up to 39%, especially during reproductive stages. The *CaHSFA5* gene, whose natural alleles regulate heat stress tolerance through reactive oxygen species (ROS) homeostasis, was recently identified in a 156.8-kb QTL region [234]. A multilocus GWAS approach identified 10 genomic regions associated with heat stress tolerance, highlighting genes, such as *RAD23b*, *CIPK25*, *AAE19*, *CK1*, and *WRKY40*. Differential expression, ROS analysis, and heterologous expression confirmed the role of these genes in heat stress regulation [235]. Danakumara *et al*. identified 27 MTAs for yield-related traits under heat stress, five of which exhibited pleiotropic effects, with SNPs near key genes such as *GH3.1* and pentatricopeptide repeat proteins, which are linked to both heat stress tolerance and yield [236]. Recently, heat shock proteins and auxin/gibberellin response factors are suggested for heat tolerance in a meta-analysis [237].

#### Pea

In pea, there are many genes conferring both salt and drought tolerance. A proline biosynthesis pathway gene *P5CR* confers drought and salt tolerance in pea [238]. Additionally, *PDH45*, a DNA helicase, seems a pleiotropic gene that has been shown to improve salt and drought tolerance, and sheath blight disease in transgenic and heterologous-overexpressed plants [239, 240]. Members of the *PsKIN* gene family were recently demonstrated to be upregulated under drought and saline stress, indicating their role as potential candidate genes [241]. For drought stress alone, the gene *PsDREB2A* may play a crucial role in pea’s response to dehydration, as its expression was upregulated in both roots and leaves of the NS MRAZ variety [242]. As a conserved gene during evolution, *COP1* from Arabidopsis regulates stomatal movements in response to dehydration in pea, providing cross-species gene resource for drought resistance [243]. Several QTLs associated with salt tolerance have been identified, though the mapping resolution is still limited [244]. Overexpression of the *PsLecRLK* gene, which encodes a lectin receptor-like kinase, in tobacco and rice, has been shown to enhance salinity tolerance by alleviating both ionic and osmotic stress and upregulating stress-responsive genes [245, 246]. Other promising candidates include *Psp68*, a salinity-induced DEAD-box protein, which enhances salt tolerance by regulating ROS and ionic balance [247].

### Biotic stress resistance

#### Common bean

In common bean, fungal diseases have been the most extensively studied [248]. For anthracnose resistance, *PvMAPK05*, *PvMAPK07*, *PvMAPK09*, and *PvMAPK11* were potentially involved in the anthracnose response, as significant changes of expression were observed in response to anthracnose infection [249]. Integrated analyses of GWAS and transcriptomic data have also identified overlapping genomic regions and common candidate genes for anthracnose resistance across multiple studies [250]. For Fusarium wilt, a methyl esterase (MES), *PvMES1*, has been identified that enhances Fusarium wilt resistance by regulating the SA-mediated signaling pathway in common bean [251]. Liu *et al*. identified eight *PvTGA* genes in common bean, distributed on six chromosomes. Among these, *PvTGA03* and *PvTGA07* have been implicated to play key roles in SA-mediated resistance to Fusarium wilt [252].

#### Faba bean

There has been relatively limited progress in mapping biotic stress resistance in faba bean. Regarding viral diseases, *Faba bean mosaic virus* (FBNYV) was reported to originate in Azerbaijan and spread to the Middle East and Africa [51]. Interestingly, the *Rhizobium leguminosarum* bv. *viciae* Strain 33 504-Mat209 has been shown to enhance faba bean resilience to *Alfalfa Mosaic Virus*. This protective effect may be attributed to reduced non-enzymatic oxidative stress indicators and increased activity of ROS scavenging enzymes, such as peroxidase (POX) and polyphenol oxidase (PPO). The transcript levels of several polyphenolic pathway genes (*C4H*, *HCT*, *C3H*, and *CHS*) and pathogenesis-related protein-1 were also elevated, suggesting their potential role in virus resistance [253]. Advances in RNA-seq have facilitated genetic mapping for resistance traits against *Ascochyta* and *Orobanche*, providing critical tools for breeding disease-resistant cultivars [254]. Furthermore, strigolactone secretion from faba bean has been shown to play a vital role in combating parasitic weeds like *Orobanche* and *Phelipanche* [255].

#### Mungbean

Bruchid resistance in mungbean is controlled by a single dominant locus, Br, with additional modifying factors. Several candidate genes for bruchid resistance have been identified near the Br locus on chromosome 5, including g5551 (encoding aspartic proteinase), g34458, and g39185 (both encoding proteins with a BURP domain) from wild mungbean [256, 257]. Further research has identified *Vradi05g03940* (*VrPGIP1*) and *Vradi05g03950* (*VrPGIP2*), both encoding polygalacturonase inhibitors, which confer resistance to bruchids [258, 259]. The *VrPGIP2* locus from mungbean landrace V2802 has been successfully utilized to enhance bruchid resistance in new mungbean varieties [260].

For Mungbean Yellow Mosaic India Virus (MYMIV), Sudha *et al*. identified the gene *Vradi04g06840*, which encodes a protein similar to suppressors of *TYPEONE PROTEINPHOSPHATASE 4MUTATION* (TOPP4–1), as a candidate for MYMIV resistance [111]. *VRADI09G06940*, a gene in the disease resistance protein family (TIR-NBS-LRR class), was recently proposed as a key candidate for MYMIV resistance [261]. In addition, MYMIV resistance has been identified in black gram. The candidate genes encoding serine–threonine kinase, UBE2D2, and BAK1/BRI1-associated receptor kinase, may provide new resources for mungbean breeding [262].

Most sources of powdery mildew (PM) resistance in mungbean are derived from Indian germplasm [263]. Fine mapping of QTLs in the immune accession RUM5 has led to the identification of the candidate gene *VrMLO12* (*LOC106773784*), which encodes a Mildew Locus O protein [264]. A cluster of *Peronospora parasitica* 13-like (NBS-LRR) genes is also considered a candidate for resistance at the qPMRUM5-2 locus [265]. Resistance to Cercospora leaf spot (CLS) has been less studied, but in the resistant germplasm V4718, CLS resistance is governed by a single gene and mapped to a stable major QTL, qCLS, located on chromosome 6. The candidate gene *LOC106765332*, which encodes a TATA-binding protein-associated factor 5 (TAF5), has been identified for CLS resistance [266].

#### Cowpea

Cowpea rust, caused by *Uromyces vignae*, is a destructive foliar disease. In 2018, Wu *et al*. identified one major QTL (Ruv1) and two minor QTLs (Ruv2 and Ruv3) conferring rust resistance in an RIL population. The Ruv2 locus was finally delimited to a 0.45-cM interval (2_09656-2_00973) through fine-mapping [267]. For Fusarium wilt, Hao *et al*. identified 31 differentially expressed *VuWRKY* genes; Four differentially expressed *WRKY* genes were selected for validation, leading to the discovery of *VuWRKY2* which can bind to the promoter region of the Catalase (*CAT*) gene, indicating its potential role in transcriptional regulation. For resistance to aphids, a transcriptome study revealed candidate resistance genes including both conventional resistance genes (e.g., LRR protein kinases), isoflavone reductases (*Vigun02g099000*, *Vigun02g099100*) using near isogenic lines [123]. For CLS resistance, Heng *et al*. fine-mapped a major QTL qCLS9.1, narrowing down to *Vigun10g019300* and *Vigun10g019400* as the candidate genes for CLS resistance in the cowpea. These genes codes for NAD-dependent malic enzyme and dynamin-related protein, respectively [268].

#### Chickpea

Some potential breeding target genes for Fusarium wilt resistance have been mapped, such as *CaFeSOD*, *CaS13like*, *CaNTAQ1*, and *CaAARS* [269]. In addition, the Transcription factors like WRKY also regulate resistance. Overexpression of *CaWRKY40* confers resistance to *Foc1* by modulating defense-related genes, while silencing *CaMPK9* increases susceptibility [270]. CaMPK9 protein protects the degradation of CaWRKY40 which induces resistance response to Fusarium wilt disease, suggesting *CaMPK9* as a novel resistance gene [270]. The *CaAHL18* gene was identified for Ascochyta blight resistance under the robust QTL qABR4.1 [271]. The flowering-associated gene, *GIGANTEA*, was identified as potentially crucial for Ascochyta blight resistance [272]. In addition, a peroxidase gene for Ascochyta blight resistance was revealed by fine mapping of qABR4.2, with higher expression in the resistant parent. The candidate gene *CaAP2* was identified [273].

#### Pea

Genes controlling resistance to Fusarium wilt have been identified, including the *Fw*, *Fwf*, and *Fnw* genes, conferring resistance to different Fop races [274, 275]. Recent studies have identified *Psat6g003960*, encoding an NB-ARC domain protein, as a candidate gene for FwS1 resistance [276]. For powdery mildew, the *Er1* (*PsMLO1*) was found to confer broad-spectrum and durable resistance in pea, with a colony abortion mechanism [277]. This gene has been deployed for breeding pea cultivars worldwide [278]. For root rot resistance, multiple QTLs have been identified [279, 280], though resistance genes remain largely unidentified. Recent studies are beginning to identify candidate genes for root rot resistance [281]. In addition, the *PDH45* gene has been confirmed for its role in expressing sheath blight disease [240].

## Applications of CIRSPR/Cas-based gene editing techniques

Although still limited in its contribution to food legume improvement, gene editing technologies, particularly CRISPR/Cas-based systems, warrant a separate section in this review. CRISPR/Cas-based genome editing has gained popularity due to its ease of construction and ability to target multiple genes simultaneously [282]. However, the application of CRISPR/Cas-based editing in legume crops remains challenging due to several factors, including genotype dependency, low editing efficiency, and poor regeneration rate [283–285]. Fortunately, hairy root transformation in legumes has enabled the rapid screening of genetically transformed lines. Food legumes have utilized sgRNAs either constructed *de novo* or borrowed from related species, such as soybean, to establish multiple editing protocols (Table 3).

**Table 3:** Recent reports of CRISPR/Cas-based gene editing in food legume crops.

**Year**	**Crop**	**Gene(s) edited**	**Validated function/improved trait**	**References**
2019	cowpea	*VuSYMRK*	symbiotic nitrogen fixation	[286]
2020	cowpea *cv*. IT86D-1010	*VuSPO11, VuREC8, VuOSD1*	meiosis	[287]
2021	common bean *cv.* Chaucha-Chuga, Ica Quimbaya, and Calima	raffinose synthase and stachyose synthase genes	indigestible polysaccharide	[288]
	chickpea (commercial kabuli)	*4CL* and *RVE7*	drought tolerance	[289]
	cowpea *cv.* IT86D-1010	*Vu-SPO11*	meiosis	[290]
2022	mungbean *var.* LGG460	AC1 and AV1 of MYMV	MYMV resistance	[291]
	pea *cv.* CDC Amarillo	*LOX*	*	[292]
	common bean	*XMPP*, *GSDA1–3*, *NSH1*, *NSH2*, *XDH*	nodule ureide biosynthesis	[293]
2023	pea *cv.* Zhongwan 6	*PsPDS*	phytoene desaturase	[294]
	pea *cv.* CDC Spectrum	*PsLOX2*	volatile organic compounds	[295]
	cowpea *var.* IT97K-499-35	*VgPDS*	phytoene desaturase	[296]
	common bean *cv.* CIAP7247F	*PvRS1, PvRS2, PvSS*	raffinose family oligosaccharides	[297]
2024	common bean *cv.* Biyuhonghua	*PvPDS*	phytoene desaturase	[298]

a Unavailable information.

Cowpea was among the earliest legume crops with gene editing using CRISPR/Cas-based technique. In 2019, the *VuSYMRK* gene encoding symbiosis receptor-like kinase (SYMRK) was mutated to show completely block of nodule formation [286]. In 2020, three cowpea meiosis genes (*SPO11-1*, *REC8*, and *OSD1*) were edited using CRISPR/Cas9 [287]. In 2021, Che *et al*. reported a rapid genome editing system using embryonic axis explants isolated from imbibed mature cowpea seeds [290]. In 2023, Bridgeland *et al*. evaluated three editing protocols in cowpea, including protoplast isolation, a transient protoplast assay, and agroinfiltration assays, by editing the *VgPDS* gene [296].

In 2023, Li *et al*. reported the first successful application of the CRISPR/Cas9 genome editing in pea by developing a transient transformation system of hairy roots. An efficient vector, PsU6.3-tRNA-PsPDS3-en35S-PsCas9, was constructed and used for editing the pea *phytoene desaturase* (*PsPDS*) gene [294]. The same year, Bhowmik *et al*. created a CRISPR construct to knock out *PsLOX2*, a gene implicated in the generation of volatile organic compounds in peas [295]. The gene-edited elite Canadian variety CDC spectrum demonstrated an improved fatty acid profile and enhanced flavor. There have been less reports of CRISPR-based editing in chickpea and mungbean. Badhan *et al*. edited the *4CL* and *RVE7* genes in chickpea protoplasts, two genes associated with drought tolerance, with high efficiency achieved for the *RVE7* gene [289]. In 2022, Pandey *et al*. used CRISPR/Cas system to edit a *LOX* gene in chickpea [292]. Talakayala *et al*. designed 20-bp sgRNAs to target the replication protein (AC1) and coat protein (AV1) genes of the MYMV genome, resulting in gene-edited mungbean with enhanced viral resistance [291].

Since 2021, there has been a surge in gene editing studies in common bean, benefiting from the high genomic similarity between common bean and soybean. Gene-editing protocols developed for soybean can be readily applied to common bean. For example, knock-out mutants of the raffinose synthase and stachyose synthase genes showed reduced levels of indigestible polysaccharides in common bean [288]. De Koning *et al*. used the hairy root transformation system to assess the efficiency of sgRNAs and the impact of different promoters. They also developed a computational model, Lindel, to accurately predict sgRNA efficiency and the type of mutation in common bean genetic transformation. De Koning *et al*. evaluated three transformation methods using Rhizobium rhizogenes K599 to induce hairy roots. They found that inoculating a severed radicle still attached to the seedling produced the highest transformation efficiency. Several highly efficient sgRNAs targeting genes involved in the biosynthesis of raffinose family oligosaccharides were identified, achieving high rates of frameshift mutations (>70%) [297]. Similarly, Wu *et al*. successfully edited the *PvPDS* gene by introducing an efficient vector, pGmUbi-Cas9-4XsgR, originally developed for soybean. This system achieved an editing efficiency exceeding 68% [298]. CRISPR/Cas9-based gene editing of *XMPP*, *GSDA1-3*, *NSH1*, *NSH2*, *XDH* genes has also been combined with metabolic analysis to validate their roles in common bean nodule ureide biosynthesis [293].

## Future perspectives

Legume research is at a critical juncture, driven by advancements in panomics. Despite these innovations, several challenges persist, ranging from the development of more applicable phenotyping systems to deeper understanding of the mechanisms underlying various stress responses. Additionally, there remains a need to efficiently leverage and pyramid elite genes for desirable traits. The following key areas are recommended as focal points for future research and improvement in food legumes.

### Better leveraging wild legume resources for genetic improvement

A current major obstacle in harnessing novel resistance genes for developing climate-resilient varieties is the under-exploration of germplasm, particularly from wild species, which may harbor unique traits that have yet to be incorporated into cultivated varieties. Over half of the germplasm in genebanks remains uncharacterized, limiting its potential for breeding [299]. Expanding and characterizing core and mini-core collections will be essential for unlocking the full genetic diversity of legumes and tapping into traits that could address current agricultural challenges. Furthermore, traits that influence consumer acceptance, such as cooking behavior (e.g. cookability) and the content of anti-nutritional factors (e.g. lectins, phytic acid), remain poorly understood, especially in wild germplasm. Mining new genes governing these traits will be critical for marketability and consumer demand.

### Harnessing ‘molecular phenomics’ for novel gene discovery

Traditionally, understanding the phenotype–genotype ties in legume yield, quality, and stress resistance have largely relied on morphological or physiological traits. However, recent technological advancements in medical phenomics, featuring full-dimensional monitoring with high accuracy and efficiency, have significantly enhanced our understanding of organism growth and development. This offers a powerful means to understanding regulatory networks from a panomics perspective and to identifying genes governing complex traits. In line with this, here we propose the adoption of ‘molecular phenomics’ into plant science, a field focused on the chemical and biochemical signatures (metabolites, proteins, transcripts, etc.) of cells and biofluids, and how these change in characteristic ways during trait onset and development. Using plant transcriptomic and metabolomic data as ‘phenotypes’ for marker-trait linkage/association analysis has already actually existed, enabling studies of expression QTLs (eQTLs), metabolite QTLs (mQTLs), expression GWAS (eGWAS), and metabolite GWAS (mGWAS). For example, Santos *et al*. quantified the expression of nine genes in chickling pea related to *Uromyces pisi* resistance and identified one cis-eQTL and one trans-eQTL controlling the expression variation of a glycosyl hydrolase family 17 gene [300].

Epigenetic diversity, a post-translational regulatory strategy, represents an additional layer of phenotypic variation. Epigenetic modifications can be developmental or acquired, depending on environmental factors, making epigenome mapping a promising avenue for uncovering dynamic regulatory mechanisms. The mapping of epigenetic quantitative trait loci (epiQTLs) has gained traction, especially in model plants like Arabidopsis [301, 302]. In crops like soybean, studies on methylQTLs [303] and the role of DNA methylation in plant-microbe interactions, including root nodulation, have already provided insights into epigenetic control [304, 305]. However, this approach has remained to be fully explored in food legumes for identifying cytosine methylation variants and key genes.

### Toward more efficient genetic manipulation in food legumes

As noted earlier, CRISPR-based gene editing in food legumes has predominantly relied on the Cas9 protein. Compared to recent advances in cereals, this gene-editing technology has lagged behind. CRISPR/Cas12 proteins have shown superior properties for *in vivo* gene editing [306]. Various Cas12 proteins, along with base editors, have been successfully adopted in rice, wheat, and other crops [307, 308], leading to significantly improved editing efficiency. Technical advances in soybean provide a useful reference for other food legumes. For example, editing of the NF-YC4 promoter in soybean resulted in higher protein content and increased fresh and dry weight in the GmNF-YC4 line [309]. We believe that continued advances in gene editing will play a crucial role in accelerating genomic studies and the development of improved legume varieties by enabling precise trait modifications.

## Data Availability

There are no new data associated with this article.

## References

[1] Lewis G, Schrire B, Mackinder B. et al. Legumes of the World. Kew, Richmond, UK: Royal Botanical Garden; 2005:592

[2] Nair R, Schreinemachers P. Global status and economic importance of mungbean. In: Nair RM, Schafleitner R, Lee S-H, eds. The Mungbean Genome. Cham: Springer International Publishing, 2020,1–8

[3] Uebersax MA, Cichy KA, Gomez FE. et al. Dry beans (*Phaseolus vulgaris* L.) as a vital component of sustainable agriculture and food security—a review. Legume Sci. 2023;5:e155

[4] Yan Z, Chu J, Nie J. et al. Legume-based crop diversification with optimal nitrogen fertilization benefits subsequent wheat yield and soil quality. Agric Ecosyst Environ. 2024;374:109171

[5] Yang X, Xiong J, Du T. et al. Diversifying crop rotation increases food production, reduces net greenhouse gas emissions and improves soil health. Nat Commun. 2024;15:198 38172570 10.1038/s41467-023-44464-9 PMC10764956

[6] Lobell DB, Burke MB, Tebaldi C. et al. Prioritizing climate change adaptation needs for food security in 2030. Science. 2008;319:607–10 18239122 10.1126/science.1152339

[7] Jain A, Sarsaiya S, Singh R. et al. Omics approaches in understanding the benefits of plant-microbe interactions. Front Microbiol. 2024;15:1391059 38860224 10.3389/fmicb.2024.1391059 PMC11163067

[8] Saxena KB, Dalvi V, Saxena RK. et al. Hybrid Breeding in Food Legumes with Special Reference to Pigeonpea, Faba bean, and Soybean. In: Saxena KB, Saxena RK, Varshney RK, eds. Genetic Enhancement in Major Food Legumes: Advances in Major Food Legumes. Springer International Publishing: Cham, 2021,123–48

[9] Varshney RK, Pandey MK, Bohra A. et al. Toward the sequence-based breeding in legumes in the post-genome sequencing era. Theor Appl Genet. 2019;132:797–816 30560464 10.1007/s00122-018-3252-x PMC6439141

[10] Weckwerth W, Ghatak A, Bellaire A. et al. PANOMICS meets germplasm. Plant Biotechnol J. 2020;18:1507–25 32163658 10.1111/pbi.13372 PMC7292548

[11] Tian Z, Nepomuceno AL, Song Q. et al. Soybean2035: a decadal vision for soybean functional genomics and breeding. Mol Plant. 2025;18:245–71 39772289 10.1016/j.molp.2025.01.004

[12] Sharma S . Prebreeding using wild species for genetic enhancement of grain legumes at ICRISAT. Crop Sci. 2017;57:1132–44

[13] Hyten DL, Song Q, Zhu Y. et al. Impacts of genetic bottlenecks on soybean genome diversity. Proc Natl Acad Sci USA. 2006;103:16666–71 17068128 10.1073/pnas.0604379103 PMC1624862

[14] Salgotra RK, Chauhan BS. Genetic diversity, conservation, and utilization of plant genetic resources. Genes. 2023;14:174 36672915 10.3390/genes14010174 PMC9859222

[15] Gideon Ladizinsky SA . The Search for Wild Relatives of Cool Season Legumes. Cham: Springer International Publishing; 2015:

[16] Rana JC, Sharma TR, Tyagi RK. et al. Characterisation of 4274 accessions of common bean (*Phaseolus vulgaris* L.) germplasm conserved in the Indian gene bank for phenological, morphological and agricultural traits. Euphytica. 2015;205:441–57

[17] Duc G, Bao S, Baum M. et al. Diversity maintenance and use of *Vicia faba* L. genetic resources. Field Crop Res. 2010;115:270–8

[18] Upadhyaya HD, Dwivedi SL, Ambrose M. et al. Legume genetic resources: management, diversity assessment, and utilization in crop improvement. Euphytica. 2011;180:27–47

[19] Commission on Genetic Resources for Food and Agriculture, FAO . The Second Report on the State of the World’s Plant Genetic Resources for Food and Agriculture. Rome: FAO; 2010

[20] Parihar AK, Kumar J, Gupta DS. et al. Genomics enabled breeding strategies for major biotic stresses in pea (*Pisum sativum* l.). Front Plant Sci. 2022;13:861191 35665148 10.3389/fpls.2022.861191 PMC9158573

[21] Pandey AK, Jiang L, Moshelion M. et al. Functional physiological phenotyping with functional mapping: a general framework to bridge the phenotype-genotype gap in plant physiology. iScience. 2021;24:102846 34381971 10.1016/j.isci.2021.102846 PMC8333144

[22] Li Y, Wu X, Xu W. et al. High-throughput physiology-based stress response phenotyping: advantages, applications and prospective in horticultural plants. Hortic Plant J. 2021;7:181–7

[23] Houle D, Govindaraju DR, Omholt S. Phenomics: the next challenge. Nat Rev Genet. 2010;11:855–66 21085204 10.1038/nrg2897

[24] Chen D, Chen M, Altmann T. et al. Bridging Genomics and Phenomics. In: Chen M, Hofestädt R, eds. Approaches in Integrative Bioinformatics: Towards the Virtual Cell. Springer Berlin Heidelberg: Berlin, Heidelberg, 2014,299–333

[25] Zhao C, Zhang Y, Du J. et al. Crop phenomics: current status and perspectives. Front Plant Sci. 2019;10:00714 10.3389/fpls.2019.00714 PMC6557228 31214228

[26] Bhandari M, Chang A, Jung J. et al. Unmanned aerial system-based high-throughput phenotyping for plant breeding. Plant Phenome J. 2023;6:e20058

[27] Burridge J, Jochua CN, Bucksch A. et al. Legume shovelomics: high-throughput phenotyping of common bean (*Phaseolus vulgaris* L.) and cowpea (*Vigna unguiculata* subsp, *unguiculata*) root architecture in the field. Field Crop Res. 2016;192:21–32

[28] Ubbens JR, Stavness I. Deep plant phenomics: a deep learning platform for complex plant phenotyping tasks. Front Plant Sci. 2017;8:01190 10.3389/fpls.2017.01190 PMC5500639 28736569

[29] Pratapa A, Doron M, Caicedo JC. Image-based cell phenotyping with deep learning. Curr Opin Chem Biol. 2021;65:9–17 34023800 10.1016/j.cbpa.2021.04.001

[30] van Dijk ADJ, Kootstra G, Kruijer W. et al. Machine learning in plant science and plant breeding. iScience. 2021;24:101890 33364579 10.1016/j.isci.2020.101890 PMC7750553

[31] Lazarević B, Carović-Stanko K, Živčak M. et al. Classification of high-throughput phenotyping data for differentiation among nutrient deficiency in common bean. Front Plant Sci. 2022;13:931877 35937354 10.3389/fpls.2022.931877 PMC9353735

[32] Wong CY, Gilbert ME, Pierce MA. et al. Hyperspectral remote sensing for phenotyping the physiological drought response of common and tepary bean. Plant Phenomics. 2023;5:0021 37040284 10.34133/plantphenomics.0021 PMC10076057

[33] Verheyen J, Dhondt S, Abbeloos R. et al. High-throughput phenotyping reveals multiple drought responses of wild and cultivated Phaseolinae beans. Front Plant Sci. 2024;15:1385985 39399541 10.3389/fpls.2024.1385985 PMC11466915

[34] Lippolis A, Polo PV, de Sousa G. et al. High-throughput seed quality analysis in faba bean: leveraging near-infrared spectroscopy (NIRS) data and statistical methods. Food Chem: X. 2024;23:101583 39071925 10.1016/j.fochx.2024.101583 PMC11282932

[35] Rane J, Raina SK, Govindasamy V. et al. Use of phenomics for differentiation of mungbean (*Vigna radiata* L. Wilczek) genotypes varying in growth rates per unit of water. Front Plant Sci. 2021;12:692564 34234800 10.3389/fpls.2021.692564 PMC8256871

[36] Vadez V, Kholová J, Hummel G. et al. LeasyScan: a novel concept combining 3D imaging and lysimetry for high-throughput phenotyping of traits controlling plant water budget. J Exp Bot. 2015;66:5581–93 26034130 10.1093/jxb/erv251 PMC4585418

[37] Fang P, Sun T, Pandey AK. et al. Understanding water conservation vs. profligation traits in vegetable legumes through a physio-transcriptomic-functional approach. Hortic Res. 2023;10:uhac287 36938572 10.1093/hr/uhac287 PMC10015340

[38] Pappula-Reddy S-P, Kumar S, Pang J. et al. High-throughput phenotyping for terminal drought stress in chickpea (*Cicer arietinum* L.). Plant Stress. 2024;11:100386

[39] Atieno J, Li Y, Langridge P. et al. Exploring genetic variation for salinity tolerance in chickpea using image-based phenotyping. Sci Rep. 2017;7:1300 28465574 10.1038/s41598-017-01211-7 PMC5430978

[40] Humplík JF, Lazár D, Fürst T. et al. Automated integrative high-throughput phenotyping of plant shoots: a case study of the cold-tolerance of pea (*Pisum sativum* L.). Plant Methods. 2015;11:20 25798184 10.1186/s13007-015-0063-9 PMC4369061

[41] Volpato L, Wright EM, Gomez FE. Drone-based digital phenotyping to evaluating relative maturity, stand count, and plant height in dry beans (*Phaseolus vulgaris* L.). Plant Phenomics. 2024;6:0278 39610705 10.34133/plantphenomics.0278 PMC11602537

[42] Ji Y, Chen Z, Cheng Q. et al. Estimation of plant height and yield based on UAV imagery in faba bean (*Vicia faba* L.). Plant Methods. 2022;18:26 35246179 10.1186/s13007-022-00861-7 PMC8897926

[43] Cui Y, Ji Y, Liu R. et al. Faba bean (*Vicia faba* L.) yield estimation based on dual-sensor data. Drones. 2023;7:378

[44] Mohammadi S, Uhlen AK, Lillemo M. et al. Enhancing phenotyping efficiency in faba bean breeding: integrating UAV imaging and machine learning. Precis Agric. 2024;25:1502–28

[45] Ji Y, Liu Z, Cui Y. et al. Faba bean and pea harvest index estimations using aerial-based multimodal data and machine learning algorithms. Plant Physiol. 2024;194:1512–26 37935623 10.1093/plphys/kiad577 PMC10904323

[46] Ji Y, Liu Z, Liu R. et al. High-throughput phenotypic traits estimation of faba bean based on machine learning and drone-based multimodal data. Comput Electron Agric. 2024;227:109584

[47] Van Haeften S, Smith D, Robinson H. et al. Unmanned aerial vehicle phenotyping of agronomic and physiological traits in mungbean. Plant Phenome J. 2025;8:e70016

[48] Zhang C, McGee RJ, Vandemark GJ. et al. Crop performance evaluation of chickpea and dry pea breeding lines across seasons and locations using phenomics data. Front Plant Sci. 2021;12:640259 33719318 10.3389/fpls.2021.640259 PMC7947363

[49] Guimarães CM, Stone LF, Zito RK. Susceptibility of common bean and soybean to water stress evaluated at the sitis phenotyping platform. Biosci J. 2017;33:871–80

[50] Rascher U, Blossfeld S, Fiorani F. et al. Non-invasive approaches for phenotyping of enhanced performance traits in bean. Funct Plant Biol. 2011;38:968–83 32480955 10.1071/FP11164

[51] Ji Y, Liu R, Xiao Y. et al. Faba bean above-ground biomass and bean yield estimation based on consumer-grade unmanned aerial vehicle RGB images and ensemble learning. Precis Agric. 2023;24:1439–60

[52] Wang J, Liu H, Ren G. Near-infrared spectroscopy (NIRS) evaluation and regional analysis of Chinese faba bean (*Vicia faba* L.). Crop J. 2014;2:28–37

[53] Johnson JB, Walsh KB, Naiker M. Assessment of bioactive compounds in faba bean using infrared spectroscopy. Legume Sci. 2023;5:e203

[54] Chiteri KO, Chiranjeevi S, Jubery TZ. et al. Dissecting the genetic architecture of leaf morphology traits in mungbean (*Vigna radiata* (L.) Wizcek) using genome-wide association study. Plant Phenome J. 2023;6:e20062

[55] Wu X, Sun T, Xu W. et al. Unraveling the genetic architecture of two complex, stomata-related drought-responsive traits by high-throughput physiological phenotyping and GWAS in cowpea (*Vigna. Unguiculata* L. Walp). Front Genet. 2021;12:743758 34777471 10.3389/fgene.2021.743758 PMC8581254

[56] Yu L, Sussman H, Khmelnitsky O. et al. Development of a mobile, high-throughput, and low-cost image-based plant growth phenotyping system. Plant Physiol. 2024;196:810–29 38696768 10.1093/plphys/kiae237

[57] Lauterberg M, Tschiersch H, Papa R. et al. Engaging precision phenotyping to scrutinize vegetative drought tolerance and recovery in chickpea plant genetic resources. Plan Theory. 2023;12:2866 10.3390/plants12152866 PMC10421427 37571019

[58] Belay AJ, Salau AO, Ashagrie M. et al. Development of a chickpea disease detection and classification model using deep learning. Inform Med Unlocked. 2022;31:100970

[59] Lauterberg M, Tschiersch H, Zhao Y. et al. Implementation of theoretical non-photochemical quenching (NPQ(T)) to investigate NPQ of chickpea under drought stress with high-throughput phenotyping. Sci Rep. 2024;14:13970 38886488 10.1038/s41598-024-63372-6 PMC11183218

[60] Sankaran S, Wang M, Vandemark GJ. Image-based rapid phenotyping of chickpeas seed size. Eng Agric Environ Food. 2016;9:50–5

[61] Zhang C, Craine WA, McGee RJ. et al. Image-based phenotyping of flowering intensity in cool-season crops. Sensors. 2020;20:1450 32155830 10.3390/s20051450 PMC7085647

[62] Bazrafkan A, Navasca H, Kim J-H. et al. Predicting dry pea maturity using machine learning and advanced sensor fusion with unmanned aerial systems (UASs). Remote Sens. 2023;15:2758

[63] Liu Z, Ji Y, Ya X. et al. Ensemble learning for pea yield estimation using unmanned aerial vehicles, red green blue, and multispectral imagery. Drones. 2024;8:227

[64] Humplík JF, Lazár D, Husičková A. et al. Automated phenotyping of plant shoots using imaging methods for analysis of plant stress responses – a review. Plant Methods. 2015;11:29 25904970 10.1186/s13007-015-0072-8 PMC4406171

[65] Schmutz J, McClean PE, Mamidi S. et al. A reference genome for common bean and genome-wide analysis of dual domestications. Nat Genet. 2014;46:707–13 24908249 10.1038/ng.3008 PMC7048698

[66] Cortinovis G, Vincenzi L, Anderson R. et al. Adaptive gene loss in the common bean pan-genome during range expansion and domestication. Nat Commun. 2024;15:6698 39107305 10.1038/s41467-024-51032-2 PMC11303546

[67] Silva DAD, Tsai SM, Chiorato AF. et al. Analysis of the common bean (*Phaseolus vulgaris* L.) transcriptome regarding efficiency of phosphorus use. PLoS One. 2019;14:e0210428 30657755 10.1371/journal.pone.0210428 PMC6338380

[68] Leitão ST, Santos C, Araújo S. et al. Shared and tailored common bean transcriptomic responses to combined fusarium wilt and water deficit. Hortic Res. 2021;8:149 34193847 10.1038/s41438-021-00583-2 PMC8245569

[69] da Silva HAP, Caetano VS, Pessôa DDV. et al. Unraveling the drought-responsive transcriptomes in nodules of two common bean genotypes during biological nitrogen fixation. Front Plant Sci. 2024;15:1345379 38344184 10.3389/fpls.2024.1345379 PMC10853390

[70] Subramani M, Urrea CA, Habib R. et al. Comparative transcriptome analysis of tolerant and sensitive genotypes of common bean (*Phaseolus vulgaris* L.) in response to terminal drought stress. Plan Theory. 2023;12:210 10.3390/plants12010210 PMC9824821 36616341

[71] Subramani M, Urrea CA, Tamatamu SR. et al. Comprehensive proteomic analysis of common bean (*Phaseolus vulgaris* l.) seeds reveal shared and unique proteins involved in terminal drought stress response in tolerant and sensitive genotypes. Biomol Ther. 2024;14:109 10.3390/biom14010109 PMC10813106 38254709

[72] Shiose L, Vidal MS, Heringer AS. et al. Proteomic analysis of common bean (*Phaseolus vulgaris* L.) leaves showed a more stable metabolism in a variety responsive to biological nitrogen fixation. Symbiosis. 2023;90:71–80

[73] Padilla-Chacón D, Campos-Patiño L, Peña-Valdivia CB. et al. Proteomic profile of tepary bean seed storage proteins in germination with low water potential. Proteome Sci. 2024;22:1 38195472 10.1186/s12953-023-00225-6 PMC10775562

[74] Cooper B, Campbell KB, Beard HS. et al. The proteomics of resistance to halo blight in common bean. Mol Plant-Microbe Interact. 2020;33:1161–75 32633604 10.1094/MPMI-05-20-0112-R

[75] Kalavacharla V . Understanding histone–DNA interactions in the common bean (*Phaseolus vulgaris* L.). Epigenetics Chromatin. 2013;6:P37

[76] Kim KD, El Baidouri M, Abernathy B. et al. A comparative epigenomic analysis of polyploidy-derived genes in soybean and common bean. Plant Physiol. 2015;168:1433–47 26149573 10.1104/pp.15.00408 PMC4528746

[77] Yung W-S, Huang C, Li M-W. et al. Changes in epigenetic features in legumes under abiotic stresses. Plant Genome. 2023;16:e20237 35730915 10.1002/tpg2.20237 PMC12807259

[78] de Lima F, Boldt ABW, Kava VM. et al. Epigenetics’ role in the common bean (*Phaseolus vulgaris* L.) and soybean (*Glycine max* (L.) Merr.) nodulation: a review. Plant Mol Biol Report. 2022;40:471–81

[79] Parker TA, Cetz J, de Sousa LL. et al. Loss of pod strings in common bean is associated with gene duplication, retrotransposon insertion and overexpression of *PvIND*. New Phytol. 2022;235:2454–65 35708662 10.1111/nph.18319

[80] Richard MMS, Gratias A, Thareau V. et al. Genomic and epigenomic immunity in common bean: the unusual features of NB-LRR gene family. DNA Res. 2018;25:161–72 29149287 10.1093/dnares/dsx046 PMC5909424

[81] Wang L, Jia G, Jiang X. et al. Altered chromatin architecture and gene expression during polyploidization and domestication of soybean. Plant Cell. 2021;33:1430–46 33730165 10.1093/plcell/koab081 PMC8254482

[82] Wu X, Chen S, Zhang Z. et al. A viral small interfering RNA-host plant mRNA pathway modulates virus-induced drought tolerance by enhancing autophagy. Plant Cell. 2024;36:3219–36 38801738 10.1093/plcell/koae158 PMC11371139

[83] Jayakodi M, Golicz AA, Kreplak J. et al. The giant diploid faba genome unlocks variation in a global protein crop. Nature. 2023;615:652–9 36890232 10.1038/s41586-023-05791-5 PMC10033403

[84] Zhao N, Zhou E, Miao Y. et al. High-quality faba bean reference transcripts generated using PacBio and Illumina RNA-seq data. Sci Data. 2024;11:359 38594303 10.1038/s41597-024-03204-4 PMC11003973

[85] Fernández-Aparicio M, Kisugi T, Xie X. et al. Low strigolactone root exudation: a novel mechanism of broomrape (*Orobanche* and *Phelipanche* spp.) resistance available for faba bean breeding. J Agric Food Chem. 2014;62:7063–71 24974726 10.1021/jf5027235

[86] Carrillo-Perdomo E, Vidal A, Kreplak J. et al. Development of new genetic resources for faba bean (*Vicia faba* L.) breeding through the discovery of gene-based SNP markers and the construction of a high-density consensus map. Sci Rep. 2020;10:6790 32321933 10.1038/s41598-020-63664-7 PMC7176738

[87] Skovbjerg CK, Angra D, Robertson-Shersby-Harvie T. et al. Genetic analysis of global faba bean diversity, agronomic traits and selection signatures. Theor Appl Genet. 2023;136:114 37074596 10.1007/s00122-023-04360-8 PMC10115707

[88] Khan MA, Alghamdi SS, Ammar MH. et al. Transcriptome profiling of faba bean (*Vicia faba* L.) drought-tolerant variety hassawi-2 under drought stress using RNA sequencing. Electron J Biotechnol. 2019;39:15–29

[89] Hou W, Zhang X, Liu Y. et al. RNA-Seq and genetic diversity analysis of faba bean (*Vicia faba* L.) varieties in China. PeerJ. 2023;11:e14259 36643650 10.7717/peerj.14259 PMC9838209

[90] Ocaña S, Seoane P, Bautista R. et al. Large-scale transcriptome analysis in Faba bean (*Vicia faba* L.) under *Ascochyta fabae* infection. PLoS One. 2015;10:e0135143 26267359 10.1371/journal.pone.0135143 PMC4534337

[91] Braich S, Sudheesh S, Forster JW. et al. Characterisation of faba bean (*Vicia faba* L.) transcriptome using RNA-Seq: sequencing, *de novo* assembly, annotation, and expression analysis. Agronomy. 2017;7:53

[92] Alghamdi SS, Khan MA, Ammar MH. et al. Characterization of drought stress-responsive root transcriptome of faba bean (*Vicia faba* L.) using RNA sequencing. 3 Biotech. 2018;8:502 10.1007/s13205-018-1518-2 PMC6258570 30498675

[93] Lyu JI, Ramekar R, Kim JM. et al. Unraveling the complexity of faba bean (*Vicia faba* L.) transcriptome to reveal cold-stress-responsive genes using long-read isoform sequencing technology. Sci Rep. 2021;11:21094 34702863 10.1038/s41598-021-00506-0 PMC8548339

[94] Björnsdotter E, Nadzieja M, Chang W. et al. VC1 catalyses a key step in the biosynthesis of vicine in faba bean. Nat Plants. 2021;7:923–31 34226693 10.1038/s41477-021-00950-w PMC7611347

[95] Yang F, Chen H, Liu C. et al. Transcriptome profile analysis of two *Vicia faba* cultivars with contrasting salinity tolerance during seed germination. Sci Rep. 2020;10:7250 32350372 10.1038/s41598-020-64288-7 PMC7190719

[96] Yuan X, Wang Q, Yan B. et al. Single-molecule real-time and illumina-based RNA sequencing data identified vernalization-responsive candidate genes in faba bean (*Vicia faba* L.). Front Genet. 2021;12:656137 34290734 10.3389/fgene.2021.656137 PMC8287337

[97] Yang SY, Habili N, Wu Q. et al. Quantitative analysis of pathway enrichment within faba bean seeds RNA-Seq (*Vicia faba* L). Am J Plant Sci. 2019;10:2305–34

[98] Shi SH, Lee SS, Zhu YM. et al. Comparative metabolomic profiling reveals key secondary metabolites associated with high quality and nutritional value in broad bean (*Vicia faba* L.). Molecules. 2022;27:8995 36558128 10.3390/molecules27248995 PMC9787534

[99] Karolkowski A, Meudec E, Bruguière A. et al. Faba bean (*Vicia faba* L. *minor*) bitterness: an untargeted metabolomic approach to highlight the impact of the non-volatile fraction. Meta. 2023;13:964 10.3390/metabo13080964 PMC10456379 37623907

[100] Karolkowski A, Belloir C, Martin C. et al. Combining sensory profiling and metabolomic approach to better understand the origins of bitter perception in faba bean (*Vicia faba L. minor*) fractions. Science Talks. 2024;11:100379

[101] Elessawy FM, Hughes J, Khazaei H. et al. A comparative metabolomics investigation of flavonoid variation in faba bean flowers. Metabolomics. 2023;19:52 37249718 10.1007/s11306-023-02014-w PMC10229742

[102] Zhang C, Ou X, Wang J. et al. Antifungal peptide P852 controls fusarium wilt in faba bean (*Vicia faba* L.) by promoting antioxidant defense and isoquinoline alkaloid, betaine, and arginine biosyntheses. Antioxidants. 2022;11:1767 36139841 10.3390/antiox11091767 PMC9495604

[103] Somta P, Laosatit K, Yuan X. et al. Thirty years of mungbean genome research: where do we stand and what have we learned? Front Plant Sci. 2022;13:944721 35909762 10.3389/fpls.2022.944721 PMC9335052

[104] Kang YJ, Kim SK, Kim MY. et al. Genome sequence of mungbean and insights into evolution within *Vigna* species. Nat Commun. 2014;5:5443 25384727 10.1038/ncomms6443 PMC4241982

[105] Kim SK, Nair RM, Lee J. et al. Genomic resources in mungbean for future breeding programs. Front Plant Sci. 2015;6:00626 10.3389/fpls.2015.00626 PMC4530597 26322067

[106] Ha J, Satyawan D, Jeong H. et al. A near-complete genome sequence of mungbean (*Vigna radiata* L.) provides key insights into the modern breeding program. Plant Genome. 2021;14:e20121 34275211 10.1002/tpg2.20121 PMC12807378

[107] Yan Q, Wang Q, Xuzhen C. et al. High-quality genome assembly, annotation and evolutionary analysis of the mungbean (*Vigna radiata*) genome. *Authorea Preprints*. 2020

[108] Liu C, Wang Y, Peng J. et al. High-quality genome assembly and pan-genome studies facilitate genetic discovery in mung bean and its improvement. Plant Commun. 2022;3:100352 35752938 10.1016/j.xplc.2022.100352 PMC9700124

[109] Jia K-H, Li G, Wang L. et al. Telomere-to-telomere, gap-free genome of mung bean (*Vigna radiata*) provides insights into domestication under structural variation. Hortic Res. 2024;;12:uhae337 40061812 10.1093/hr/uhae337 PMC11886820

[110] Liu C, Fan B, Cao Z. et al. A deep sequencing analysis of transcriptomes and the development of EST-SSR markers in mungbean (*Vigna radiata*). J Genet. 2016;95:527–35 27659323 10.1007/s12041-016-0663-9

[111] Sudha M, Karthikeyan A, Madhumitha B. et al. Dynamic transcriptome profiling of mungbean genotypes unveil the genes respond to the infection of mungbean yellow mosaic virus. Pathogens. 2022;11:190 35215133 10.3390/pathogens11020190 PMC8874377

[112] Kumar S, Ayachit G, Sahoo L. Screening of mungbean for drought tolerance and transcriptome profiling between drought-tolerant and susceptible genotype in response to drought stress. Plant Physiol Biochem. 2020;157:229–38 33129069 10.1016/j.plaphy.2020.10.021

[113] Kazłowski B, Chen M-R, Chao P-M. et al. Identification and roles of proteins for seed development in mungbean (*Vigna radiata* L.) seed proteomes. J Agric Food Chem. 2013;61:6650–9 23758297 10.1021/jf401170g

[114] Wu X, Wang Y, Tang H. Quantitative metabonomic analysis reveals the germination-associated dynamic and systemic biochemical changes for mung-bean (*Vigna radiata*) seeds. J Proteome Res. 2020;19:2457–70 32393034 10.1021/acs.jproteome.0c00181

[115] Kang YJ, Bae A, Shim S. et al. Genome-wide DNA methylation profile in mungbean. Sci Rep. 2017;7:40503 28084412 10.1038/srep40503 PMC5233969

[116] Ha J, Kwon H, Cho KH. et al. Identification of epigenetic variation associated with synchronous pod maturity in mungbean (*Vigna radiata* L.). Sci Rep. 2020;10:17414 33060755 10.1038/s41598-020-74520-z PMC7562708

[117] Zhao P, Ma B, Cai C. et al. Transcriptome and methylome changes in two contrasting mungbean genotypes in response to drought stress. BMC Genomics. 2022;23:80 35078408 10.1186/s12864-022-08315-z PMC8790888

[118] Lonardi S, Muñoz-Amatriaín M, Liang Q. et al. The genome of cowpea (*Vigna unguiculata* [L.] Walp.). Plant J. 2019;98:767–82 31017340 10.1111/tpj.14349 PMC6852540

[119] Xia Q, Pan L, Zhang R. et al. The genome assembly of asparagus bean, *Vigna unguiculata* ssp. *sesquipedialis*. Sci Data. 2019;6:124 31316072 10.1038/s41597-019-0130-6 PMC6638192

[120] Pan L, Liu M, Kang Y. et al. Comprehensive genomic analyses of *Vigna unguiculata* provide insights into population differentiation and the genetic basis of key agricultural traits. Plant Biotechnol J. 2023;21:1426–39 36965079 10.1111/pbi.14047 PMC10281604

[121] Liang Q, Muñoz-Amatriaín M, Shu S. et al. A view of the pan-genome of domesticated cowpea (*Vigna unguiculata* [L.] Walp.). Plant Genome. 2024;17:e20319 36946261 10.1002/tpg2.20319 PMC12807118

[122] Wu X, Hu Z, Zhang Y. et al. Differential selection of yield and quality traits has shaped genomic signatures of cowpea domestication and improvement. Nat Genet. 2024;56:992–1005 38649710 10.1038/s41588-024-01722-w

[123] MacWilliams JR, Nabity PD, Mauck KE. et al. Transcriptome analysis of aphid-resistant and susceptible near isogenic lines reveals candidate resistance genes in cowpea (*Vigna unguiculata*). BMC Plant Biol. 2023;23:22 36631779 10.1186/s12870-022-04021-w PMC9832699

[124] Abiala M, Sadhukhan A, Muthuvel J. et al. Rhizosphere *Priestia* species altered cowpea root transcriptome and enhanced growth under drought and nutrient deficiency. Planta. 2022;257:11 36515736 10.1007/s00425-022-04047-2

[125] Barrera-Figueroa BE, Gao L, Diop NN. et al. Identification and comparative analysis of drought-associated microRNAs in two cowpea genotypes. BMC Plant Biol. 2011;11:127 21923928 10.1186/1471-2229-11-127 PMC3182138

[126] Martins TF, Souza PFN, Alves MS. et al. Identification, characterization, and expression analysis of cowpea (*Vigna unguiculata* [L.] Walp.) miRNAs in response to cowpea severe mosaic virus (CPSMV) challenge. Plant Cell Rep. 2020;39:1061–78 32388590 10.1007/s00299-020-02548-6

[127] Paul S, Kundu A, Pal A. Identification and validation of conserved microRNAs along with their differential expression in roots of *Vigna unguiculata* grown under salt stress. Plant Cell Tissue Organ Cult. 2011;105:233–42

[128] Bai C, Zhang F, Meng D. et al. Sodium nitroprusside (SNP) generated nitric oxide delays senescence of cowpea (*Vigna unguiculata* (L.) Walp). Postharvest Biol Technol. 2024;214:112976

[129] Liu J, Wei L, Zhu L. et al. Integrative transcriptome and metabolome analyses reveal the mechanism of melatonin in delaying postharvest senescence in cowpeas. Int J Biol Macromol. 2024;282:137429 39528182 10.1016/j.ijbiomac.2024.137429

[130] Zhang S, Yin F, Li J. et al. Transcriptomic and metabolomic investigation of metabolic disruption in *Vigna unguiculata* L. triggered by acetamiprid and cyromazine. Ecotoxicol Environ Saf. 2022;239:113675 35617907 10.1016/j.ecoenv.2022.113675

[131] Tsamo AT, Mohammed H, Mohammed M. et al. Seed coat metabolite profiling of cowpea (*Vigna unguiculata* L. Walp.) accessions from Ghana using UPLC-PDA-QTOF-MS and chemometrics. Nat Prod Res. 2020;34:1158–62 30663354 10.1080/14786419.2018.1548463

[132] Li T, Feng M, Chi Y. et al. Defensive resistance of cowpea *Vigna unguiculata* control *Megalurothrips usitatus* mediated by jasmonic acid or insect damage. Plan Theory. 2023;12:942 10.3390/plants12040942 PMC9967092 36840292

[133] Han L, Wang Z, Wang Q. et al. Multiomics comprehensive analysis of pre-storage low-temperature on cowpea metabolism. Postharvest Biol Technol. 2024;216:113056

[134] Varshney RK, Song C, Saxena RK. et al. Draft genome sequence of chickpea (*Cicer arietinum*) provides a resource for trait improvement. Nat Biotechnol. 2013;31:240–6 23354103 10.1038/nbt.2491

[135] Rehman SA, Gul S, Parthiban M. et al. Genetic resources and genes/QTLs for gram pod borer (*Helicoverpa armigera* Hübner) resistance in chickpea from the Western Himalayas. Plant Genome. 2024;17:e20483 38965817 10.1002/tpg2.20483 PMC12807025

[136] Roorkiwal M, Jain A, Kale SM. et al. Development and evaluation of high-density Axiom®Cicer Array for high-resolution genetic mapping and breeding applications in chickpea. Plant Biotechnol J. 2018;16:890–901 28913885 10.1111/pbi.12836 PMC5866945

[137] Varshney RK, Roorkiwal M, Sun S. et al. A chickpea genetic variation map based on the sequencing of 3,366 genomes. Nature. 2021;599:622–7 34759320 10.1038/s41586-021-04066-1 PMC8612933

[138] Khan AW, Garg V, Sun S. et al. *Cicer* super-pangenome provides insights into species evolution and agronomic trait loci for crop improvement in chickpea. Nat Genet. 2024;56:1225–34 38783120 10.1038/s41588-024-01760-4

[139] Negussu M, Karalija E, Vergata C. et al. Drought tolerance mechanisms in chickpea (*Cicer arietinum* L.) investigated by physiological and transcriptomic analysis. Environ Exp Bot. 2023;215:105488

[140] Basso MF, Girardin G, Vergata C. et al. Genome-wide transcript expression analysis reveals major chickpea and lentil genes associated with plant branching. Front Plant Sci. 2024;15:1384237 38962245 10.3389/fpls.2024.1384237 PMC11220206

[141] Gupta S, Mishra SK, Misra S. et al. Revealing the complexity of protein abundance in chickpea root under drought-stress using a comparative proteomics approach. Plant Physiol Biochem. 2020;151:88–102 32203884 10.1016/j.plaphy.2020.03.005

[142] Vessal S, Arefian M, Siddique KHM. Proteomic responses to progressive dehydration stress in leaves of chickpea seedlings. BMC Genomics. 2020;21:523 32727351 10.1186/s12864-020-06930-2 PMC7392671

[143] Chaturvedi P, Pierides I, López-Hidalgo C. et al. Natural variation in the chickpea metabolome under drought stress. Plant Biotechnol J. 2024;22:3278–94 39411896 10.1111/pbi.14447 PMC11606430

[144] Raman R, Morris S, Sharma N. et al. Metabolite profiling of chickpea (*Cicer arietinum*) in response to necrotrophic fungus *Ascochyta rabiei*. Front Plant Sci. 2024;15:1427688 39193211 10.3389/fpls.2024.1427688 PMC11347347

[145] Singh V, Gupta K, Singh S. et al. Unravelling the molecular mechanism underlying drought stress response in chickpea *via* integrated multi-omics analysis. Front Plant Sci. 2023;14:1156606 37287713 10.3389/fpls.2023.1156606 PMC10242046

[146] Kudapa H, Ghatak A, Barmukh R. et al. Integrated multi-omics analysis reveals drought stress response mechanism in chickpea (*Cicer arietinum* L.). Plant Genome. 2024;17:e20337 37165696 10.1002/tpg2.20337 PMC12806944

[147] Daware A, Mohanty JK, Narnoliya L. et al. Uncovering DNA methylation landscapes to decipher evolutionary footprints of phenotypic diversity in chickpea. DNA Res. 2024;31:dsae013 38702947 10.1093/dnares/dsae013 PMC11149376

[148] Pradhan S, Verma S, Chakraborty A. et al. Identification and molecular characterization of miRNAs and their target genes associated with seed development through small RNA sequencing in chickpea. Funct Integr Genom. 2021;21:283–98 10.1007/s10142-021-00777-w 33630193

[149] Ucar S, Yaprak E, Yigider E. et al. Genome-wide analysis of miR172-mediated response to heavy metal stress in chickpea (*Cicer arietinum* L.): physiological, biochemical, and molecular insights. BMC Plant Biol. 2024;24:1063 39528933 10.1186/s12870-024-05786-y PMC11555882

[150] Priyadarshini P, Kalwan G, Kohli D. et al. Small RNA sequencing analysis provides novel insights into microRNA-mediated regulation of defense responses in chickpea against Fusarium wilt infection. Planta. 2025;261:23 39751997 10.1007/s00425-024-04599-5

[151] Tiwari M, Singh B, Yadav M. et al. High throughput identification of miRNAs reveal novel interacting targets regulating chickpea-rhizobia symbiosis. Environ Exp Bot. 2021;186:104469

[152] Sindhu A, Ramsay L, Sanderson L-A. et al. Gene-based SNP discovery and genetic mapping in pea. Theor Appl Genet. 2014;127:2225–41 25119872 10.1007/s00122-014-2375-y PMC4180032

[153] Pandey AK, Rubiales D, Wang Y. et al. Omics resources and omics-enabled approaches for achieving high productivity and improved quality in pea (*Pisum sativum* L.). Theor Appl Genet. 2021;134:755–76 33433637 10.1007/s00122-020-03751-5

[154] Chen H, Osuna D, Colville L. et al. Transcriptome-wide mapping of pea seed ageing reveals a pivotal role for genes related to oxidative stress and programmed cell death. PLoS One. 2013;8:e78471 24205239 10.1371/journal.pone.0078471 PMC3812160

[155] Kreplak J, Madoui M-A, Cápal P. et al. A reference genome for pea provides insight into legume genome evolution. Nat Genet. 2019;51:1411–22 31477930 10.1038/s41588-019-0480-1

[156] Yang T, Liu R, Luo Y. et al. Improved pea reference genome and pan-genome highlight genomic features and evolutionary characteristics. Nat Genet. 2022;54:1553–63 36138232 10.1038/s41588-022-01172-2 PMC9534762

[157] Liu N, Lyu X, Zhang X. et al. Reference genome sequence and population genomic analysis of peas provide insights into the genetic basis of Mendelian and other agronomic traits. Nat Genet. 2024;56:1964–74 39103648 10.1038/s41588-024-01867-8

[158] Jan N, Rather AM-U-D, John R. et al. Proteomics for abiotic stresses in legumes: present status and future directions. Crit Rev Biotechnol. 2023;43:171–90 35109728 10.1080/07388551.2021.2025033

[159] Mamontova T, Lukasheva E, Mavropolo-Stolyarenko G. et al. Proteome map of pea (*Pisum sativum* L.) embryos containing different amounts of residual chlorophylls. Int J Mol Sci. 2018;19:4066 30558315 10.3390/ijms19124066 PMC6320946

[160] Daba SD, Panda P, Aryal UK. et al. Proteomics analysis of round and wrinkled pea (*Pisum sativum* L.) seeds during different development periods. Proteomics. 2025;25:e2300363 39475056 10.1002/pmic.202300363 PMC11794676

[161] Reinprecht Y, Qi Y, Shahmir F. et al. Yield and antiyield genes in common bean (*Phaseolus vulgaris* L.). Legume Sci. 2021;3:e91

[162] Xu K, Zhu J, Zhai H. et al. A single-nucleotide polymorphism in *PvPW1* encoding β-1,3-glucanase 9 is associated with pod width in *Phaseolus vulgaris* L. J Genet Genomics. 2024;51:1413–22 39389459 10.1016/j.jgg.2024.09.020

[163] Aragão FJL, Barros LMG, Sousa MV. et al. Expression of a methionine-rich storage albumin from the Brazil nut (*Bertholletia excelsa* H.B.K., Lecythidaceae) in transgenic bean plants (*Phaseolus vulgaris* L., Fabaceae). Genet Mol Biol. 1999;22:445–9

[164] Astudillo-Reyes C, Fernandez AC, Cichy KA. Transcriptome characterization of developing bean (P*haseolus vulgaris* l.) pods from two genotypes with contrasting seed zinc concentrations. PLoS One. 2015;10:e0137157 26367119 10.1371/journal.pone.0137157 PMC4569411

[165] de Koning R, Kiekens R, Toili MEM. et al. Identification and expression analysis of the genes involved in the raffinose family oligosaccharides pathway of *Phaseolus vulgaris* and *Glycine max*. Plan Theory. 2021;10:1465 10.3390/plants10071465 PMC8309293 34371668

[166] Yilmaz H, Özer G, Baloch FS. et al. Genome-wide identification and expression analysis of MTP (metal ion transport proteins) genes in the common bean. Plan Theory. 2023;12:3218 10.3390/plants12183218 PMC10535811 37765382

[167] Montanini B, Blaudez D, Jeandroz S. et al. Phylogenetic and functional analysis of the cation diffusion facilitator (CDF) family: improved signature and prediction of substrate specificity. BMC Genomics. 2007;8:107 17448255 10.1186/1471-2164-8-107 PMC1868760

[168] Perez de Souza L, Scossa F, Proost S. et al. Multi-tissue integration of transcriptomic and specialized metabolite profiling provides tools for assessing the common bean (*Phaseolus vulgaris*) metabolome. Plant J. 2019;97:1132–53 30480348 10.1111/tpj.14178 PMC6850281

[169] Zhao N, Xue D, Miao Y. et al. Construction of a high-density genetic map for faba bean (*Vicia faba* L.) and quantitative trait loci mapping of seed-related traits. Front Plant Sci. 2023;14:1201103 37351218 10.3389/fpls.2023.1201103 PMC10282779

[170] Ohm H, Åstrand J, Ceplitis A. et al. Novel SNP markers for flowering and seed quality traits in faba bean (*Vicia faba* L.): characterization and GWAS of a diversity panel. Front Plant Sci. 2024;15:1348014 38510437 10.3389/fpls.2024.1348014 PMC10950902

[171] Gutierrez N, Pégard M, Solis I. et al. Genome-wide association study for yield-related traits in faba bean (*Vicia faba* L.). Front Plant Sci. 2024;15:1328690 38545396 10.3389/fpls.2024.1328690 PMC10965552

[172] Webb A, Cottage A, Wood T. et al. A SNP-based consensus genetic map for synteny-based trait targeting in faba bean (*Vicia faba* L.). Plant Biotechnol J. 2016;14:177–85 25865502 10.1111/pbi.12371 PMC4973813

[173] Gutierrez N, Torres AM. Characterization and diagnostic marker for *TTG1* regulating tannin and anthocyanin biosynthesis in faba bean. Sci Rep. 2019;9:16174 31700069 10.1038/s41598-019-52575-x PMC6838129

[174] Gutierrez N, Avila CM, Torres AM. The bHLH transcription factor VfTT8 underlies zt2, the locus determining zero tannin content in faba bean (*Vicia faba* L.). Sci Rep. 2020;10:14299 32868815 10.1038/s41598-020-71070-2 PMC7459296

[175] Liu J, Lin Y, Chen J. et al. Genome-wide association studies provide genetic insights into natural variation of seed-size-related traits in mungbean. Front Plant Sci. 2022;13:997988 36311130 10.3389/fpls.2022.997988 PMC9608654

[176] Han X, Li L, Chen H. et al. Resequencing of 558 Chinese mungbean landraces identifies genetic loci associated with key agronomic traits. Front Plant Sci. 2022;13:1043784 36311125 10.3389/fpls.2022.1043784 PMC9597495

[177] Lin Y, Laosatit K, Liu J. et al. The mungbean *VrP* locus encoding MYB90, an R2R3-type MYB protein, regulates anthocyanin biosynthesis. Front Plant Sci. 2022;13:895634 35937322 10.3389/fpls.2022.895634 PMC9355716

[178] Wang Q, Cao H, Wang J. et al. Fine-mapping and primary analysis of candidate genes associated with seed coat color in mung bean (*Vigna radiata* L.). J Integr Agric. 2024;23:2571–88

[179] Ma C, Feng Y, Zhou S. et al. Metabolomics and transcriptomics provide insights into the molecular mechanisms of anthocyanin accumulation in the seed coat of differently colored mung bean (*Vigna radiata* L.). Plant Physiol Biochem. 2023;200:107739 37196373 10.1016/j.plaphy.2023.107739

[180] Watcharatpong P, Kaga A, Chen X. et al. Narrowing down a major QTL region conferring pod fiber contents in Yardlong bean (*Vigna unguiculata*), a vegetable cowpea. Genes. 2020;11:363 32230893 10.3390/genes11040363 PMC7230914

[181] Wu X, Michael VN, López-Hernández F. et al. Genetic diversity and genome-wide association in cowpeas (*Vigna unguiculata* L. Walp). Agronomy. 2024;14:961

[182] Lo S, Muñoz-Amatriaín M, Hokin SA. et al. A genome-wide association and meta-analysis reveal regions associated with seed size in cowpea [*Vigna unguiculata* (L.) Walp]. Theor Appl Genet. 2019;132:3079–87 31367839 10.1007/s00122-019-03407-z PMC6791911

[183] Yang Y, Wu Z, Wu Z. et al. A near-complete assembly of asparagus bean provides insights into anthocyanin accumulation in pods. Plant Biotechnol J. 2023;21:2473–89 37558431 10.1111/pbi.14142 PMC10651155

[184] Li Y, Chen Q, Xie X. et al. Integrated metabolomics and transcriptomics analyses reveal the molecular mechanisms underlying the accumulation of anthocyanins and other flavonoids in cowpea pod (*Vigna unguiculata* L.). J Agric Food Chem. 2020;68:9260–75 32709199 10.1021/acs.jafc.0c01851

[185] Wang R, Gangola MP, Irvine C. et al. Co-localization of genomic regions associated with seed morphology and composition in a desi chickpea (*Cicer arietinum* L.) population varying in seed protein concentration. Theor Appl Genet. 2019;132:1263–81 30661107 10.1007/s00122-019-03277-5

[186] Upadhyaya HD, Bajaj D, Narnoliya L. et al. Genome-wide scans for delineation of candidate genes regulating seed-protein content in chickpea. Front Plant Sci. 2016;7:00302 10.3389/fpls.2016.00302 PMC4803732 27047499

[187] Chakraborty A, Junaid A, Parida SK. et al. Integrated genomic approaches delineate a novel role of *ROP1 ENHANCER1* in controlling seed protein content of chickpea. J Exp Bot. 2023;74:817–34 36378574 10.1093/jxb/erac452

[188] Sab S, Lokesha R, Mannur DM. et al. Genome-wide SNP discovery and mapping QTLs for seed iron and zinc concentrations in chickpea (*Cicer arietinum* L.). Front Nutr. 2020;7:559120 33154975 10.3389/fnut.2020.559120 PMC7588353

[189] Singh G, Ambreen H, Jain P. et al. Comparative transcriptomic and metabolite profiling reveals genotype-specific responses to Fe starvation in chickpea. Physiol Plant. 2023;175:e13897 36960640 10.1111/ppl.13897

[190] Tan GZH, Das Bhowmik SS, Hoang TML. et al. Investigation of baseline iron levels in Australian chickpea and evaluation of a transgenic biofortification approach. Front Plant Sci. 2018;9:00788 10.3389/fpls.2018.00788 PMC6010650 29963065

[191] Cheng P, Holdsworth W, Ma Y. et al. Association mapping of agronomic and quality traits in USDA pea single-plant collection. Mol Breed. 2015;35:75

[192] Gali KK, Sackville A, Tafesse EG. et al. Genome-wide association mapping for agronomic and seed quality traits of field pea (*Pisum sativum* L.). Front Plant Sci. 2019;10:01538 10.3389/fpls.2019.01538 PMC6888555 31850030

[193] Ma Y, Coyne CJ, Grusak MA. et al. Genome-wide SNP identification, linkage map construction and QTL mapping for seed mineral concentrations and contents in pea (*Pisum sativum* L.). BMC Plant Biol. 2017;17:43 28193168 10.1186/s12870-016-0956-4 PMC5307697

[194] Chen B, Shi Y, Sun Y. et al. Innovations in functional genomics and molecular breeding of pea: exploring advances and opportunities. aBIOTECH. 2024;5:71–93 38576433 10.1007/s42994-023-00129-1 PMC10987475

[195] Rayner T, Moreau C, Ambrose M. et al. Genetic variation controlling wrinkled seed phenotypes in *Pisum*: how lucky was Mendel? Int J Mol Sci. 2017;18:1205 28587311 10.3390/ijms18061205 PMC5486028

[196] Clemente A, Arques MC, Dalmais M. et al. Eliminating anti-nutritional plant food proteins: the case of seed protease inhibitors in pea. PLoS One. 2015;10:e0134634 26267859 10.1371/journal.pone.0134634 PMC4534040

[197] Zhang P, Wang Y, Sun T. et al. Fine mapping *PsPS1*, a gene controlling pod softness that defines market type in pea (*Pisum sativum*). Plant Breed. 2022;141:418–28

[198] Villordo-Pineda E, González-Chavira MM, Giraldo-Carbajo P. et al. Identification of novel drought-tolerant-associated SNPs in common bean (*Phaseolus vulgaris*). Front Plant Sci. 2015;6:00546 10.3389/fpls.2015.00546 PMC4508514 26257755

[199] Cortés AJ, Blair MW. Genotyping by sequencing and genome–environment associations in wild common bean predict widespread divergent adaptation to drought. Front Plant Sci. 2018;9:00128 10.3389/fpls.2018.00128 PMC5826387 29515597

[200] López CM, Pineda M, Alamillo JM. Transcriptomic response to water deficit reveals a crucial role of phosphate acquisition in a drought-tolerant common bean landrace. Plan Theory. 2020;9:445 10.3390/plants9040445 PMC7238123 32252433

[201] Wu L, Chang Y, Wang L. et al. The aquaporin gene *PvXIP1;2* conferring drought resistance identified by GWAS at seedling stage in common bean. Theor Appl Genet. 2022;135:485–500 34698878 10.1007/s00122-021-03978-w

[202] Dong X, Zhu H, Hao X. et al. Genome-wide identification of common bean *PvLTP* family genes and expression profiling analysis in response to drought stress. Genes. 2022;13:2394 36553661 10.3390/genes13122394 PMC9777604

[203] Hiz MC, Canher B, Niron H. et al. Transcriptome analysis of salt tolerant common bean (*Phaseolus vulgaris* L.) under saline conditions. PLoS One. 2014;9:e92598 24651267 10.1371/journal.pone.0092598 PMC3961409

[204] Niron H, Türet M. A putative common bean chalcone *O*-methyltransferase improves salt tolerance in transgenic *Arabidopsis thaliana*. J Plant Growth Regul. 2020;39:957–69

[205] González AM, Yuste-Lisbona FJ, Weller J. et al. Characterization of QTL and environmental interactions controlling flowering time in Andean common bean (*Phaseolus vulgaris* L.). Front Plant Sci. 2021;11:599462 33519852 10.3389/fpls.2020.599462 PMC7840541

[206] Vargas Y, Mayor-Duran VM, Buendia HF. et al. Physiological and genetic characterization of heat stress effects in a common bean RIL population. PLoS One. 2021;16:e0249859 33914759 10.1371/journal.pone.0249859 PMC8084131

[207] Chavarro MC, Blair MW. QTL analysis and effect of the *fin* locus on tropical adaptation in an inter-gene pool common bean population. Trop Plant Biol. 2010;3:204–18

[208] Suárez JC, Polanía JA, Contreras AT. et al. Adaptation of common bean lines to high temperature conditions: genotypic differences in phenological and agronomic performance. Euphytica. 2020;216:28

[209] Ammar MH, Khan AM, Migdadi HM. et al. Faba bean drought responsive gene identification and validation. Saudi J Biol Sci. 2017;24:80–9 28053575 10.1016/j.sjbs.2016.05.011 PMC5199002

[210] Huang L-T, Liu C-Y, Li L. et al. Genome-wide identification of bZIP transcription factors in faba bean based on transcriptome analysis and investigation of their function in drought response. Plan Theory. 2023;12:3041 10.3390/plants12173041 PMC10490193 37687286

[211] Cao Y-Y, Bian X-C, Chen M-X. et al. iTRAQ-based quantitative proteomic analysis in vernalization-treated faba bean (*Vicia faba* L.). PLoS One. 2017;12:e0187436 29121109 10.1371/journal.pone.0187436 PMC5679601

[212] Maalouf F, Abou-Khater L, Babiker Z. et al. Genetic dissection of heat stress tolerance in faba bean (*Vicia faba* L.) using GWAS. Plan Theory. 2022;11:1108 10.3390/plants11091108 PMC9103424 35567109

[213] Chang Y, Peng L, Ji L. et al. Genome-wise association study identified genomic regions associated with drought tolerance in mungbean (*Vigna radiata* (L.) R. Wilczek). Theor Appl Genet. 2023;136:40 36897414 10.1007/s00122-023-04303-3

[214] Lin Y, Amkul K, Laosatit K. et al. Fine mapping of QTL conferring resistance to calcareous soil in mungbean reveals *VrYSL3* as candidate gene for the resistance. Plant Sci. 2023;332:111698 37028455 10.1016/j.plantsci.2023.111698

[215] Liu J, Xue C, Lin Y. et al. Genetic analysis and identification of *VrFRO8*, a salt tolerance-related gene in mungbean. Gene. 2022;836:146658 35714797 10.1016/j.gene.2022.146658

[216] Breria CM, Hsieh CH, Yen TB. et al. A SNP-based genome-wide association study to mine genetic loci associated to salinity tolerance in mungbean (*Vigna radiata* L.). Genes. 2020;11:759 32646058 10.3390/genes11070759 PMC7397256

[217] Li S, Liu J, Xue C. et al. Identification and functional characterization of WRKY, PHD and MYB three salt stress responsive gene families in mungbean (*Vigna radiata* L.). Genes. 2023;14:463 36833390 10.3390/genes14020463 PMC9956968

[218] Wu R, Chen J, Lin Y. et al. Genome-wide identification, expression analysis, and potential roles under abiotic stress of the *YUCCA* gene family in mungbean (*Vigna radiata* L.). Int J Mol Sci. 2023;24:1603 36675117 10.3390/ijms24021603 PMC9866024

[219] Wu R, Jia Q, Guo Y. et al. Characterization of TBP and TAFs in mungbean (*Vigna radiata* L.) and their potential involvement in abiotic stress response. Int J Mol Sci. 2024;25:9558 39273505 10.3390/ijms25179558 PMC11394781

[220] Li G, Wu X, Hu Y. et al. Orphan genes are involved in drought adaptations and ecoclimatic-oriented selections in domesticated cowpea. J Exp Bot. 2019;70:3101–10 30949664 10.1093/jxb/erz145

[221] Sadhukhan A, Kobayashi Y, Kobayashi Y. et al. VuDREB2A, a novel DREB2-type transcription factor in the drought-tolerant legume cowpea, mediates DRE-dependent expression of stress-responsive genes and confers enhanced drought resistance in transgenic *Arabidopsis*. Planta. 2014;240:645–64 25030652 10.1007/s00425-014-2111-5

[222] Chankaew S, Isemura T, Naito K. et al. QTL mapping for salt tolerance and domestication-related traits in *Vigna marina* subsp. *oblonga*, a halophytic species. Theor Appl Genet. 2014;127:691–702 24370961 10.1007/s00122-013-2251-1

[223] Ravelombola W, Shi A, Weng Y. et al. Association analysis of salt tolerance in cowpea (*Vigna unguiculata* (L.) Walp) at germination and seedling stages. Theor Appl Genet. 2018;131:79–91 28948303 10.1007/s00122-017-2987-0

[224] Ravelombola W, Shi A, Huynh B-L. et al. Genetic architecture of salt tolerance in a Multi-Parent Advanced Generation Inter-Cross (MAGIC) cowpea population. BMC Genomics. 2022;23:100 35123403 10.1186/s12864-022-08332-y PMC8817504

[225] Pan L, Yu X, Shao J. et al. Transcriptomic profiling and analysis of differentially expressed genes in asparagus bean (*Vigna unguiculata* ssp. *sesquipedalis*) under salt stress. PLoS One. 2019;14:e0219799 31299052 10.1371/journal.pone.0219799 PMC6625716

[226] Srivastava R, Kobayashi Y, Koyama H. et al. Cowpea NAC1/NAC2 transcription factors improve growth and tolerance to drought and heat in transgenic cowpea through combined activation of photosynthetic and antioxidant mechanisms. J Integr Plant Biol. 2023;65:25–44 36107155 10.1111/jipb.13365

[227] Mishra S, Sahu G, Shaw BP. Insight into the cellular and physiological regulatory modulations of class-I TCP9 to enhance drought and salinity stress tolerance in cowpea. Physiol Plant. 2022;174:e13542 34459503 10.1111/ppl.13542

[228] Liang L, Sui X, Xiao J. et al. ERD14 regulation by the HY5- or HY5-MED2 module mediates the cold signal transduction of asparagus bean. Plant J. 2025;121:e17172 39589925 10.1111/tpj.17172

[229] Liang L, Wang D, Xu D. et al. Comparative phylogenetic analysis of the mediator complex subunit in asparagus bean (*Vigna unguiculata* ssp. *sesquipedialis*) and its expression profile under cold stress. BMC Genomics. 2024;25:149 38321384 10.1186/s12864-024-10060-4 PMC10848533

[230] Yadava YK, Chaudhary P, Yadav S. et al. Genetic mapping of quantitative trait loci associated with drought tolerance in chickpea (*Cicer arietinum* L.). Sci Rep. 2023;13:17623 37848483 10.1038/s41598-023-44990-y PMC10582051

[231] Thudi M, Samineni S, Li W. et al. Whole genome resequencing and phenotyping of MAGIC population for high resolution mapping of drought tolerance in chickpea. Plant Genome. 2024;17:e20333 37122200 10.1002/tpg2.20333 PMC12806880

[232] Istanbuli T, Nassar AE, Abd El-Maksoud MM. et al. Genome-wide association study reveals SNP markers controlling drought tolerance and related agronomic traits in chickpea across multiple environments. Front Plant Sci. 2024;15:1260690 38525151 10.3389/fpls.2024.1260690 PMC10957531

[233] Ahmed SM, Alsamman AM, Jighly A. et al. Genome-wide association analysis of chickpea germplasms differing for salinity tolerance based on DArTseq markers. PLoS One. 2021;16:e0260709 34852014 10.1371/journal.pone.0260709 PMC8635330

[234] Mohanty JK, Thakro V, Yadav A. et al. Delineation of genes for a major QTL governing heat stress tolerance in chickpea. Plant Mol Biol. 2024;114:19 38363401 10.1007/s11103-024-01421-4

[235] Mohanty JK, Yadav A, Narnoliya L. et al. A next-generation combinatorial genomic strategy scans genomic loci governing heat stress tolerance in chickpea. Plant Cell Environ. 2025;48:2706–26 39360859 10.1111/pce.15186

[236] Danakumara T, Kumar N, Patil BS. et al. Unraveling the genetics of heat tolerance in chickpea landraces (*Cicer arietinum* L.) using genome-wide association studies. Front Plant Sci. 2024;15:1376381 38590753 10.3389/fpls.2024.1376381 PMC10999645

[237] Panigrahi S, Kumar U, Swami S. et al. Meta QTL analysis for dissecting abiotic stress tolerance in chickpea. BMC Genomics. 2024;25:439 38698307 10.1186/s12864-024-10336-9 PMC11067088

[238] Farooq M, Ahmad R, Shahzad M. et al. Real-time expression and in silico characterization of pea genes involved in salt and water-deficit stress. Mol Biol Rep. 2023;51:18 38099977 10.1007/s11033-023-09064-2

[239] Sahoo RK, Chandan RK, Swain DM. et al. Heterologous overexpression of *PDH45* gene of pea provides tolerance against sheath blight disease and drought stress in rice. Plant Physiol Biochem. 2022;186:242–51 35930936 10.1016/j.plaphy.2022.07.018

[240] Shivakumara TN, Sreevathsa R, Dash PK. et al. Overexpression of pea DNA helicase 45 (*PDH45*) imparts tolerance to multiple abiotic stresses in chili (*Capsicum annuum* L.). Sci Rep. 2017;7:2760 28584274 10.1038/s41598-017-02589-0 PMC5459802

[241] Yuan H, Liu B, Zhang G. et al. Genome-wide identification and expression analysis of the *PsKIN* gene family in pea. Front Genet. 2024;15:1510864 39744065 10.3389/fgene.2024.1510864 PMC11689205

[242] Jovanovic Z, Stanisavljevic N, Mikic A. et al. The expression of drought responsive element binding protein (*DREB2A*) related gene from pea (*Pisum sativum* L.) as affected by water stress. Aust J Crop Sci. 2013;7:1590–6

[243] Moazzam-Jazi M, Ghasemi S, Seyedi SM. et al. COP1 plays a prominent role in drought stress tolerance in Arabidopsis and pea. Plant Physiol Biochem. 2018;130:678–91 30139551 10.1016/j.plaphy.2018.08.015

[244] Jha UC, Bohra A, Jha R. et al. Salinity stress response and ‘omics’ approaches for improving salinity stress tolerance in major grain legumes. Plant Cell Rep. 2019;38:255–77 30637478 10.1007/s00299-019-02374-5

[245] Vaid N, Pandey P, Srivastava VK. et al. Pea lectin receptor-like kinase functions in salinity adaptation without yield penalty, by alleviating osmotic and ionic stresses and upregulating stress-responsive genes. Plant Mol Biol. 2015;88:193–206 25863480 10.1007/s11103-015-0319-9

[246] Passricha N, Saifi SK, Kharb P. et al. Marker-free transgenic rice plant overexpressing pea *LecRLK* imparts salinity tolerance by inhibiting sodium accumulation. Plant Mol Biol. 2019;99:265–81 30604324 10.1007/s11103-018-0816-8

[247] Banu MSA, Huda KMK, Sahoo RK. et al. Pea p68 imparts salinity stress tolerance in rice by scavenging of ROS-mediated H_2_O_2_ and interacts with argonaute. Plant Mol Biol Report. 2015;33:221–38

[248] Jain P, Singh S, Sinha S. et al. Chapter 17 - Common bean disease improvement using QTL mapping. In: Wani SH, Wang D, Pratap Singh G, eds. QTL Mapping in Crop Improvement. New York, USA: Academic Press; 2023:355–76

[249] Liu H, Wang D, Wang Z. et al. Identification of MAPK genes in *Phaseolus vulgaris* and analysis of their expression patterns in response to anthracnose. Int J Mol Sci. 2024;25:13101 39684810 10.3390/ijms252313101 PMC11641984

[250] Shafi S, Tahir M, Bawa V. et al. Biochemical defense arsenal, genes/QTLs and transcripts for imparting anthracnose resistance in common bean (*Phaseolus vulgaris* L.). Plant Stress. 2024;14:100609

[251] Xue R, Feng M, Chen J. et al. A methyl esterase 1 (*PvMES1*) promotes the salicylic acid pathway and enhances Fusarium wilt resistance in common beans. Theor Appl Genet. 2021;134:2379–98 34128089 10.1007/s00122-021-03830-1

[252] Liu Y, Huang Y, Li Z. et al. Genome-wide identification of the *TGA* genes in common bean (*Phaseolus vulgaris*) and revealing their functions in response to *Fusarium oxysporum* f. sp. *phaseoli* infection. Front Genet. 2023;14:1137634 36755571 10.3389/fgene.2023.1137634 PMC9901207

[253] Abdelkhalek A, Bashir S, El-Gendi H. et al. Protective activity of *Rhizobium leguminosarum* bv. *Viciae* strain 33504-mat209 against alfalfa mosaic virus infection in faba bean plants. Plan Theory. 2023;12:2658 10.3390/plants12142658 PMC10384385 37514271

[254] Rubiales D, Rojas-Molina MM, Sillero JC. Characterization of resistance mechanisms in faba bean (*Vicia faba*) against broomrape species (*Orobanche* and *Phelipanche* spp.). Front Plant Sci. 2016;7:1747 27920790 10.3389/fpls.2016.01747 PMC5118618

[255] Fernández-Aparicio M, Yoneyama K, Rubiales D. The role of strigolactones in host specificity of *Orobanche* and *Phelipanche* seed germination. Seed Sci Res. 2011;21:55–61

[256] Lin W-J, Ko C-Y, Liu M-S. et al. Transcriptomic and proteomic research to explore bruchid-resistant genes in mungbean isogenic lines. J Agric Food Chem. 2016;64:6648–58 27508985 10.1021/acs.jafc.6b03015

[257] Liu M-S, Kuo TC-Y, Ko C-Y. et al. Genomic and transcriptomic comparison of nucleotide variations for insights into bruchid resistance of mungbean (*Vigna radiata* [L.] R. Wilczek). BMC Plant Biol. 2016;16:46 26887961 10.1186/s12870-016-0736-1 PMC4756517

[258] Chotechung S, Somta P, Chen J. et al. A gene encoding a polygalacturonase-inhibiting protein (PGIP) is a candidate gene for bruchid (Coleoptera: bruchidae) resistance in mungbean (*Vigna radiata*). Theor Appl Genet. 2016;129:1673–83 27220975 10.1007/s00122-016-2731-1

[259] Kaewwongwal A, Liu C, Somta P. et al. A second *VrPGIP1* allele is associated with bruchid resistance (*Callosobruchus* spp.) in wild mungbean (*Vigna radiata* var. *sublobata*) accession ACC41. Mol Gen Genomics. 2020;295:275–86 10.1007/s00438-019-01619-y 31705195

[260] Wu R, Zhang Q, Lin Y. et al. Marker-assisted backcross breeding for improving bruchid (*Callosobruchus* spp.) resistance in mung bean (*Vigna radiata* L.). Agronomy. 2022;12:1271

[261] Kohli M, Bansal H, Mishra GP. et al. Genome-wide association studies for earliness, MYMIV resistance, and other associated traits in mungbean (*Vigna radiata* L. Wilczek) using genotyping by sequencing approach. PeerJ. 2024;12:e16653 38288464 10.7717/peerj.16653 PMC10823994

[262] Dhaliwal SK, Gill RK, Sharma A. et al. A large-effect QTL introgressed from ricebean imparts resistance to *Mungbean yellow mosaic India virus* in blackgram (*Vigna mungo* (L.) Hepper). Theor Appl Genet. 2022;135:4495–506 36271056 10.1007/s00122-022-04234-5

[263] Chankaew S, Somta P, Isemura T. et al. Quantitative trait locus mapping reveals conservation of major and minor loci for powdery mildew resistance in four sources of resistance in mungbean [*Vigna radiata* (L.) Wilczek]. Mol Breed. 2013;32:121–30

[264] Yundaeng C, Somta P, Chen J. et al. Candidate gene mapping reveals *VrMLO12* (*MLO* clade II) is associated with powdery mildew resistance in mungbean (*Vigna radiata* [L.] Wilczek). Plant Sci. 2020;298:110594 32771151 10.1016/j.plantsci.2020.110594

[265] Waengwan P, Laosatit K, Lin Y. et al. A cluster of *peronospora parasitica 13-like* (*NBS-LRR*) genes is associated with powdery mildew (*Erysiphe polygoni*) resistance in mungbean (*Vigna radiata*). Plan Theory. 2024;13:1230 10.3390/plants13091230 PMC11085486 38732445

[266] Yundaeng C, Somta P, Chen J. et al. Fine mapping of QTL conferring Cercospora leaf spot disease resistance in mungbean revealed TAF5 as candidate gene for the resistance. Theor Appl Genet. 2021;134:701–14 33188437 10.1007/s00122-020-03724-8

[267] Wu X, Li G, Wang B. et al. Fine mapping *Ruv2*, a new rust resistance gene in cowpea (*Vigna unguiculata*), to a 193-kb region enriched with NBS-type genes. Theor Appl Genet. 2018;131:2709–18 30225641 10.1007/s00122-018-3185-4

[268] Heng T, Kaga A, Chen X. et al. Two tightly linked genes coding for NAD-dependent malic enzyme and dynamin-related protein are associated with resistance to Cercospora leaf spot disease in cowpea (*Vigna unguiculata* (L.) Walp.). Theor Appl Genet. 2020;133:395–407 31691838 10.1007/s00122-019-03470-6

[269] Alsamman AM, Mousa KH, Istanbuli T. et al. Unveiling the genetic basis of Fusarium wilt resistance in chickpea using GWAS analysis and characterization of candidate genes. Front Genet. 2024;14:1292009 38327700 10.3389/fgene.2023.1292009 PMC10849131

[270] Chakraborty J, Ghosh P, Sen S. et al. CaMPK9 increases the stability of CaWRKY40 transcription factor which triggers defense response in chickpea upon *Fusarium oxysporum* f. sp. *ciceri* Race1 infection. Plant Mol Biol. 2019;100:411–31 30953279 10.1007/s11103-019-00868-0

[271] Kumar K, Purayannur S, Kaladhar VC. et al. mQTL-seq and classical mapping implicates the role of an *AT-HOOK MOTIF CONTAINING NUCLEAR LOCALIZED* (*AHL*) family gene in *Ascochyta* blight resistance of chickpea. Plant Cell Environ. 2018;41:2128–40 29492990 10.1111/pce.13177

[272] Alo F, Rani AR, Baum M. et al. Novel genomic regions linked to *Ascochyta* blight resistance in two differentially resistant cultivars of chickpea. Front Plant Sci. 2022;13:762002 35548283 10.3389/fpls.2022.762002 PMC9083910

[273] Singh R, Kumar K, Purayannur S. et al. Genomics-assisted genetics of complex regions from chickpea chromosome 4 reveals two candidate genes for *Ascochyta* blight resistance. Plant Sci. 2023;334:111781 37392939 10.1016/j.plantsci.2023.111781

[274] Jain S, Weeden NF, Kumar A. et al. Functional codominant marker for selecting the *Fw* gene conferring resistance to Fusarium wilt race 1 in pea. Crop Sci. 2015;55:2639–46

[275] Mc Phee KE, Inglis DA, Gundersen B. et al. Mapping QTL for Fusarium wilt race 2 partial resistance in pea (*Pisum sativum*). Plant Breed. 2012;131:300–6

[276] Deng D, Sun S, Wu W. et al. Fine mapping and identification of a Fusarium wilt resistance gene *FwS1* in pea. Theor Appl Genet. 2024;137:171 38918246 10.1007/s00122-024-04682-1

[277] Humphry M, ReinstÄDler A, Ivanov S. et al. Durable broad-spectrum powdery mildew resistance in pea *er1* plants is conferred by natural loss-of-function mutations in *PsMLO1*. Mol Plant Pathol. 2011;12:866–78 21726385 10.1111/j.1364-3703.2011.00718.x PMC6640514

[278] Iglesias-García R, Rubiales D, Fondevilla S. Penetration resistance to *Erysiphe pisi* in pea mediated by *er1* gene is associated with protein cross-linking but not with callose apposition or hypersensitive response. Euphytica. 2015;201:381–7

[279] Jha AB, Gali KK, Alam Z. et al. Potential application of genomic technologies in breeding for fungal and oomycete disease resistance in pea. Agronomy. 2021;11:1260

[280] Leprévost T, Boutet G, Lesné A. et al. Advanced backcross QTL analysis and comparative mapping with RIL QTL studies and GWAS provide an overview of QTL and marker haplotype diversity for resistance to Aphanomyces root rot in pea (*Pisum sativum*). Front Plant Sci. 2023;14:1189289 37841625 10.3389/fpls.2023.1189289 PMC10569610

[281] Kälin C, Piombo E, Bourras S. et al. Transcriptomic analysis identifies candidate genes for Aphanomyces root rot disease resistance in pea. BMC Plant Biol. 2024;24:144 38413860 10.1186/s12870-024-04817-y PMC10900555

[282] Singh C, Kumar R, Sehgal H. et al. Unclasping potentials of genomics and gene editing in chickpea to fight climate change and global hunger threat. Front Genet. 2023;14:1085024 37144131 10.3389/fgene.2023.1085024 PMC10153629

[283] Hnatuszko-Konka K, Kowalczyk T, Gerszberg A. et al. *Phaseolus vulgaris*—recalcitrant potential. Biotechnol Adv. 2014;32:1205–15 24953179 10.1016/j.biotechadv.2014.06.001

[284] Ma X, Zhu Q, Chen Y. et al. CRISPR/Cas9 platforms for genome editing in plants: developments and applications. Mol Plant. 2016;9:961–74 27108381 10.1016/j.molp.2016.04.009

[285] Li C, Chu W, Gill RA. et al. Computational tools and resources for CRISPR/Cas genome editing. Genomics Proteomics Bioinformatics. 2023;21:108–26 35341983 10.1016/j.gpb.2022.02.006 PMC10372911

[286] Ji J, Zhang C, Sun Z. et al. Genome editing in cowpea *Vigna unguiculata* using CRISPR-Cas9. Int J Mol Sci. 2019;20:2471 31109137 10.3390/ijms20102471 PMC6566367

[287] Juranić M, Nagahatenna DSK, Salinas-Gamboa R. et al. A detached leaf assay for testing transient gene expression and gene editing in cowpea (*Vigna unguiculata* [L.] Walp.). Plant Methods. 2020;16:88 32549904 10.1186/s13007-020-00630-4 PMC7296760

[288] Fernando A, Selvaraj M, Chavarriaga P. et al. A clearinghouse for genome-edited crops and field testing. Mol Plant. 2021;14:3–5 33340689 10.1016/j.molp.2020.12.010

[289] Badhan S, Ball AS, Mantri N. First report of CRISPR/Cas9 mediated DNA-free editing of *4CL* and *RVE7* genes in chickpea protoplasts. Int J Mol Sci. 2021;22:396 33401455 10.3390/ijms22010396 PMC7795094

[290] Che P, Chang S, Simon MK. et al. Developing a rapid and highly efficient cowpea regeneration, transformation and genome editing system using embryonic axis explants. Plant J. 2021;106:817–30 33595147 10.1111/tpj.15202 PMC8252785

[291] Talakayala A, Mekala GK, Reddy MK. et al. Manipulating resistance to mungbean yellow mosaic virus in greengram (*Vigna radiata* L): through CRISPR/Cas9 mediated editing of the viral genome. Front Sustain Food Syst. 2022;6:911574

[292] Pandey PK, Bhowmik P, Kagale S. Optimized methods for random and targeted mutagenesis in field pea (*Pisum sativum* L.). Front Plant Sci. 2022;13:995542 36160971 10.3389/fpls.2022.995542 PMC9498975

[293] Voß L, Heinemann KJ, Herde M. et al. Enzymes and cellular interplay required for flux of fixed nitrogen to ureides in bean nodules. Nat Commun. 2022;13:5331 36088455 10.1038/s41467-022-33005-5 PMC9464200

[294] Li G, Liu R, Xu R. et al. Development of an *Agrobacterium*-mediated CRISPR/Cas9 system in pea (*Pisum sativum* L.). Crop J. 2023;11:132–9

[295] Bhowmik P, Yan W, Hodgins C. et al. CRISPR/Cas9-mediated lipoxygenase gene-editing in yellow pea leads to major changes in fatty acid and flavor profiles. Front Plant Sci. 2023;14:1246905 37810390 10.3389/fpls.2023.1246905 PMC10552856

[296] Bridgeland A, Biswas S, Tsakirpaloglou N. et al. Optimization of gene editing in cowpea through protoplast transformation and agroinfiltration by targeting the phytoene desaturase gene. PLoS One. 2023;18:e0283837 37018323 10.1371/journal.pone.0283837 PMC10075407

[297] de Koning R, Daryanavard H, Garmyn J. et al. Fine-tuning CRISPR/Cas9 gene editing in common bean (*Phaseolus vulgaris* L.) using a hairy root transformation system and *in silico* prediction models. Front Plant Sci. 2023;14:1233418 37929181 10.3389/fpls.2023.1233418 PMC10623320

[298] Wu X, Zhang P, Chen S. et al. A molecular toolkit to boost functional genomic studies in transformation-recalcitrant vegetable legumes. Hortic Res. 2023;10:uhad064 37249953 10.1093/hr/uhad064 PMC10208893

[299] Tripathi K, Gore PG, Singh MF. et al. Legume genetic resources: status and opportunities for sustainability. In: Hasanuzzaman M, ed. Legume Crops - Prospects, Production and Uses. IntechOpen:London, UK; 2020:1–10

[300] Santos C, Martins DC, González-Bernal MJ. et al. Integrating phenotypic and gene expression linkage mapping to dissect rust resistance in chickling pea. Front Plant Sci. 2022;13:837613 35463408 10.3389/fpls.2022.837613 PMC9021875

[301] Furci L, Jain R, Stassen J. et al. Identification and characterisation of hypomethylated DNA loci controlling quantitative resistance in *Arabidopsis*. elife. 2019;8:e40655 30608232 10.7554/eLife.40655 PMC6342528

[302] Pankaj R, Shoejaeyfar S, Figueiredo DD. An epiQTL underlying asexual seed formation in Arabidopsis. Plant Reprod. 2024;37:463–8 38836892 10.1007/s00497-024-00504-y PMC11511731

[303] Schmitz RJ, He Y, Valdés-López O. et al. Epigenome-wide inheritance of cytosine methylation variants in a recombinant inbred population. Genome Res. 2013;23:1663–74 23739894 10.1101/gr.152538.112 PMC3787263

[304] Satgé C, Moreau S, Sallet E. et al. Reprogramming of DNA methylation is critical for nodule development in *Medicago truncatula*. Nat Plants. 2016;2:16166 27797357 10.1038/nplants.2016.166

[305] Niyikiza D, Piya S, Routray P. et al. Interactions of gene expression, alternative splicing, and DNA methylation in determining nodule identity. Plant J. 2020;103:1744–66 32491251 10.1111/tpj.14861

[306] Badon IW, Oh Y, Kim H-J. et al. Recent application of CRISPR-Cas12 and OMEGA system for genome editing. Mol Ther. 2024;32:32–43 37952084 10.1016/j.ymthe.2023.11.013 PMC10787141

[307] Lv P, Su F, Chen F. et al. Genome editing in rice using CRISPR/Cas12i3. Plant Biotechnol J. 2024;22:379–85 37822083 10.1111/pbi.14192 PMC10826996

[308] Wang W, Yan L, Li J. et al. Engineering a robust Cas12i3 variant-mediated wheat genome editing system. Plant Biotechnol J. 2025;23:860–73 39690508 10.1111/pbi.14544 PMC11869199

[309] Wang L, O'Conner S, Tanvir R. et al. CRISPR/Cas9-based editing of *NF-YC4* promoters yields high-protein rice and soybean. New Phytol. 2024;245:2103–16 39307530 10.1111/nph.20141 PMC11798907

